# Updated systematic review of current randomised controlled trials in chronic subdural haematoma

**DOI:** 10.1007/s00701-025-06683-5

**Published:** 2025-11-06

**Authors:** R. Fakhry, C. Yesildal, J. Bartek, J. Duerinck, T. S. R. Jensen, J. Soleman, C. Iorio-Morin, C. M. F. Dirven, R. Dammers, E. Edlmann, D. C. Holl, on be half of the International Collaborative Research Initiative on Chronic Subdural Haematoma (iCORIC) study group

**Affiliations:** 1 Department of Neurosurgery, Erasmus Medical Center, Erasmus MC Stroke Center, Rotterdam, the Netherlands; 2 Department of Neurosurgery, Karolinska University Hospital, Stockholm, Sweden; 3 Department of Neurosurgery, Universitair Ziekenhuis Brussel, Vrije Universiteit Brussel, Brussels, Belgium; 4 Department of Neurosurgery, Copenhagen University Hospital, Copenhagen, Denmark; 5 Department of Neurosurgery, University Hospital Basel, Basel, Switzerland; 6 Division of Neurosurgery, Department of Surgery, Centre Hospitalier Universitaire de Sherbrooke, Sherbrooke, Canada; 7 Department of Neurosurgery, South West Neurosurgical Centre, Plymouth, United Kingdom; 8 Department of Clinical Neuroscience, Karolinska Institutet, Solna, Sweden

**Keywords:** CSDH, RCTs, BHC, MMAE, Corticosteroids, RoB 2

## Abstract

**Background:**

Chronic subdural haematoma (CSDH) is a common neurosurgical condition with an increasing incidence due to an aging population. Given the expanding research landscape, assessing the state of recent trials is essential. This systematic review updates a previous review, which included randomised controlled trials (RCTs) up to 2019, by summarizing recently published and ongoing RCTs in CSDH, highlighting key areas of investigation and identifying directions for future research.

**Methods:**

Clinical trial registries – including the Cochrane Controlled Register of Trials, WHO ICTRP, clinicaltrials.gov, and Clinical Trials Information System – were systematically searched for RCTs on CSDH from June 1, 2019, to February 18, 2025. Both published and ongoing trials were included in this review.

**Results:**

This review identified 41 recently published RCTs and 54 ongoing RCTs, compared to 26 ongoing trials in 2019. Of the earlier review, eleven studies have been published, five remain active, and the remainder were either abandoned or did not adhere to their initial RCT design. Middle meningeal artery embolisation (MMAE) has become the most extensively studied intervention, with active trials increasing from 2 in 2019 to 21 in 2025. Trials investigating perioperative management (3 versus 7) and surgical techniques (5 versus 10) have also increased. In contrast, corticosteroid trials have decreased (7 versus 3), likely reflecting findings from recent high-impact studies. Research on tranexamic acid has increased (5 versus 7) as have studies on other pharmacological agents (4 versus 8).

**Conclusions:**

The number of ongoing RCTs in CSDH has increased substantially, with a notable shift in research focus. MMAE now dominates the field, though the surge in studies may suggest research saturation. Future investigations may benefit from more collaborative efforts, consolidating resources into fewer, but larger and adequately powered trials.

**Supplementary Information:**

The online version contains supplementary material available at 10.1007/s00701-025-06683-5.

## Introduction

Chronic subdural haematoma (CSDH) is one of the most common neurosurgical conditions. It occurs most frequently in elderly patients, with an incidence that continues to rise due to the aging population [5, 9, 90, 104, 119]. Despite its prevalence, there is currently no consensus on the optimal treatment strategy for CSDH.

The current management of CSDH involves a broad range of treatment options. Surgical intervention, particularly burr hole evacuation with postoperative drainage, remains the most widely used approach for symptomatic CSDH [15, 40, 93, 111]. However, variations in surgical technique persist, not only globally but also at national and even intrahospital levels [12, 13, 50, 67]. These variations include, among other factors, the type and duration of postoperative drainage, the choice between general or local anesthesia, and the number of burr holes used. Alternative treatment strategies may also be considered depending on the patient’s clinical presentation. These may include different surgical interventions, such as craniotomy, pharmacological therapies, middle meningeal artery embolisation (MMAE), or watchful waiting. The publication of recent clinical practice guidelines represents an important step toward standardizing CSDH care [106, 118]. These guidelines, however, acknowledge the limited evidence supporting both optimal surgical and non-surgical approaches, emphasising the need for further high-quality research to address these gaps.

**Fig. 1:**
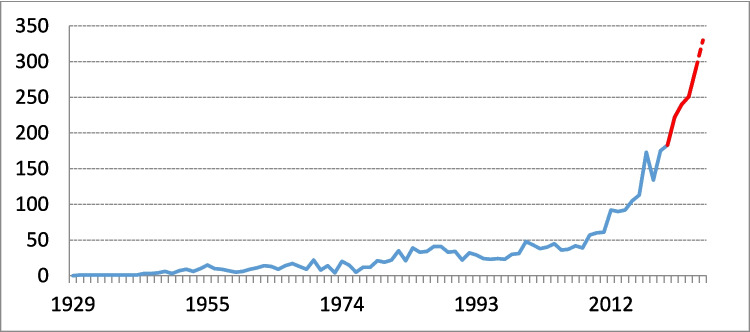
Evolution of the number of publications per year on chronic subdural haematoma from 1929 to 2025, based on title-specific search terms: (chronic) AND (subdural) AND (hematomas OR haematomas OR hemorrhages OR haemorrhages OR hematoma OR haematoma OR hemorrhage OR bleeding). The red segment marks recent years (2020–2025), representing the period since the previous review. The dashed line indicates the number of publications for 2025, extrapolated based on trends from previous years

To address these evidence gaps, randomised controlled trials (RCTs) play a crucial role in advancing evidence-based care for CSDH. A systematic review conducted in 2019 by the international COllaborative Research Initiative on Chronic subdural haematoma (iCORIC) identified 26 active RCTs investigating treatment approaches for CSDH [35]. This review highlighted the growing interest in improving CSDH outcomes and emphasized the need for future RCTs to guide clinical decision-making. In the years since, this research field has continued to expand exponentially, reflected by a significant increase in the number of CSDH-related publications (Fig. 1).

Importantly, several of the RCTs identified in the 2019 review have now been completed, providing valuable insights into the field and have already been translated into clinical practice. For example, trials on steroids as either an adjunct or alternative to surgery in CSDH demonstrated inferior outcomes for treatment with dexamethasone [53, 76]. This has led to a significant move away from steroid use in CSDH, which is reflected in clinical practice guidelines in some countries [59, 118]. This demonstrates the significant and rapid influence well-designed RCTs can have on clinical practice and in shaping future research directions. Alternative pharmacological agents such as tranexamic acid and statins are now more of a research focus, alongside the minimally invasive MMAE.

Considering the progress made in CSDH therapies since 2019 and the emergence of new studies, this systematic update of RCTs in CSDH is aimed to help inform both clinicians and researchers of current evidence in this field. Critical evaluation of recently completed and ongoing RCTs will provide a comprehensive overview of the therapeutic landscape, identify overlap between studies, and propose directions for future research to address persistent knowledge gaps.

## Methods

This systematic review was conducted in accordance with the Preferred Reporting Items for Systematic Reviews and Meta-Analyses (PRISMA) 2020 statement [96]. The review was prospectively registered in the PROSPERO register under the identifier CRD42024604895.

Four trial registries were systematically searched from June 1, 2019 to February 18, 2025: the Cochrane Controlled Register of Trials (CENTRAL), the World Health Organization International Clinical Trials Registry Platform (WHO ICTRP), clinicaltrials.gov, and Clinical Trials Information System (CTIS). The search strategy from the 2019 review was updated and is detailed in Table 1.

**Table 1:** Search terms for trials databases

Cochrane CENTRAL	WHO ICTRP	Clinicaltrials.gov	CTIS (separate searches)
([mh "Hematoma, Subdural, Chronic"] OR (Chronic* NEAR/3 ((subdural*) NEAR/3 (hematom* OR haematom* OR hemorrhag* OR haemorrhag* OR bleeding*))):ab,ti,kw)	subdural AND hematoma OR subdural AND haematoma OR subdural AND hemorrhage OR subdural AND haemorrhage OR subdural AND bleeding	((subdural) AND (hematoma OR haematoma OR hemorrhage OR haemorrhage OR bleeding))	- subdural hematoma - subdural haematoma - subdural hemorrhage - subdural haemorrhage - subdural bleeding

Two independent reviewers (RF, DCH) screened the titles and abstracts of all identified trials published from June 1, 2019 up to February 18, 2025. Trials were excluded if they were duplicates, did not focus on CSDH, or were not an RCT by design. Both ongoing and published trials were included if a summary was available in English. Any disagreements between the reviewers were resolved through discussion until consensus was reached.

The final list of identified trials was further categorized into two groups: published trials and ongoing trials. Trials that appeared in both groups were removed from the ‘ongoing’ category. However, if only interim results had been published and the trial was still ongoing, it remained in the ‘ongoing’ category and was excluded from the ‘published’ group. The trials included in the 2019 review were cross-referenced with the new set of trials to ensure overlap and to categorize them as either ‘published’ or ‘ongoing’, based on their current status.

Ongoing trials underwent further review to verify their status. This process involved electronic searches of research groups and trial titles, as well as email communication with registered principal investigators (PIs) to assess trial progress and estimated completion dates. Trials were excluded if they were explicitly reported as abandoned or if there was no response from the PI and the estimated completion date was at least three years prior to the search.

The methodology of all published trials was assessed using the Cochrane Risk of Bias 2 (RoB 2) tool [116]. One reviewer (RF) conducted the initial assessment of all trials. In cases of uncertainty, the final judgment was reached through discussion and consensus with two additional reviewers (DCH, EE).

## Results

**Fig. 2:**
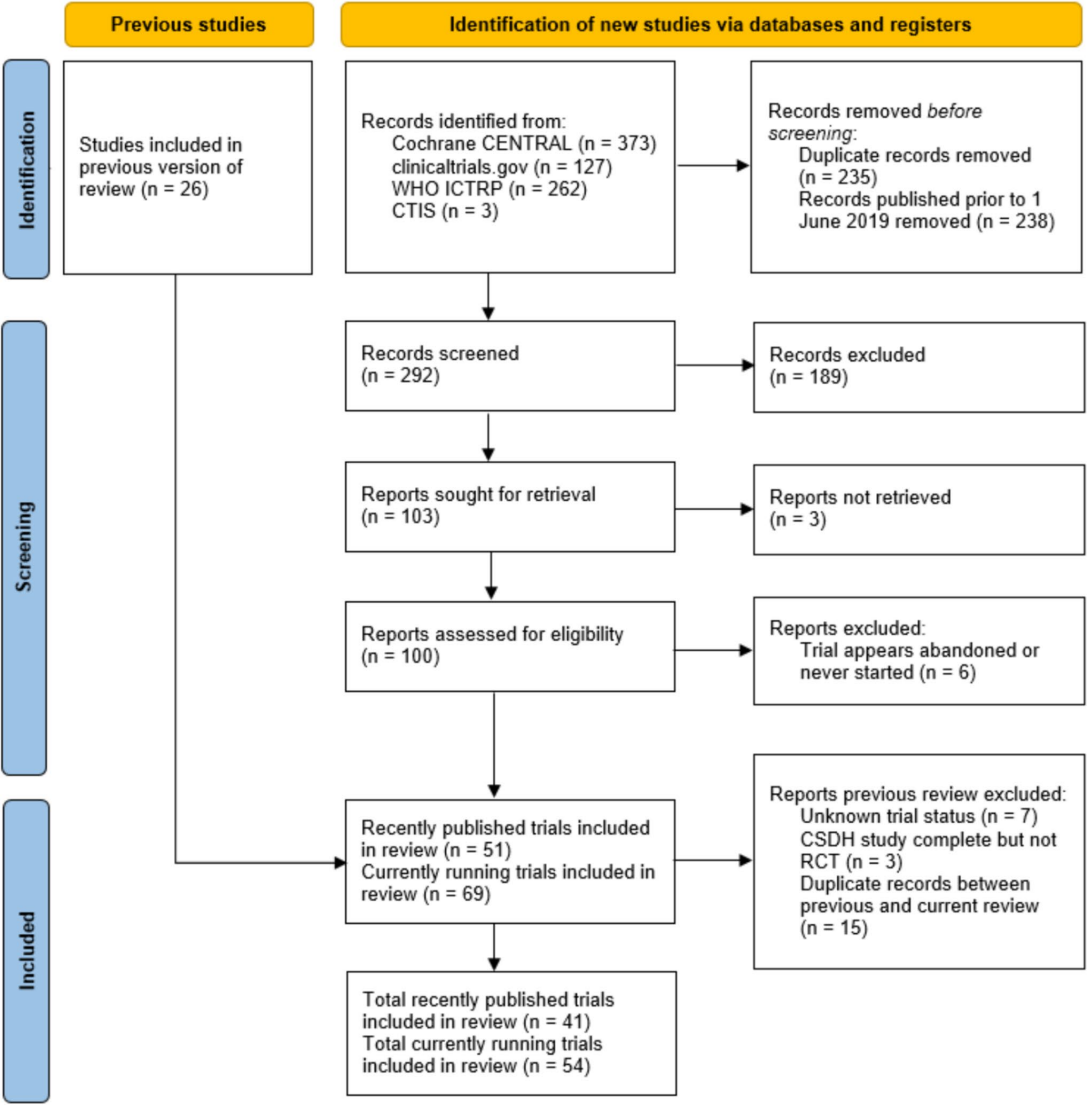
Flowchart of the identification, screening and inclusion of RCTs in this review

A total of 291 eligible records were identified and screened for inclusion following the search strategy. After initial screening, 100 articles met the inclusion criteria. Of these, 6 trials were either apparently abandoned or never initiated, leaving 94 studies for further analysis. Cross-referencing with ongoing trials identified in the 2019 review led to the inclusion of one additional study, bringing the final total to 95 trials. These were categorized as published trials (*n* = 41) and ongoing trials (*n* = 54) (Fig. 2). Among the published trials, eleven had been identified in the previous review, along with five of the ongoing trials.

The RCT topics were categorized into the most common trial themes; steroids, tranexamic acid, other pharmacological agents, surgical techniques, perioperative management, MMA embolisation, and miscellaneous trials. The published and ongoing trials are presented in Tables 2 and 3, respectively.

**Table 2:** Overview of all recently published RCTs in CSDH included in review

Reference	Trial name; title	Primary objective	Year	Country	Trial design	N	RoB	Patients	Intervention	Comparison	Outcome (primary)	Conclusions
**Steroid trials = 5**												
P. J. Hutchinson et al. [53]	Dex-CSDH; DXM for CSDH	To assess the effect of a 2-week tapering course of DXM on functional outcomes in patients with symptomatic CSDH	2020	United Kingdom	Multicenter, double-blind, placebo-controlled RCT	748	Low risk	Patients ≥ 18 years with symptomatic CSDH, predominantly iso- or hypodense on imaging	DXM for 14-day tapering course; starting with 16 mg/day for 3 days, reducing every 3 days	Placebo as per DXM regimen	Modified Rankin Scale at 6 months	In adults with symptomatic CSDH, most of whom underwent surgical evacuation during the index admission, treatment with DXM resulted in fewer favorable outcomes and more adverse events than placebo at 6 months, but resulted in fewer repeat operations
I. P. Miah et al. [76]	DECSA; DXM versus surgery for CSDH	To investigate noninferiority of primary DXM therapy versus BHC on functional outcome	2023	Netherlands	Multicenter, noninferiority RCT with PROBE design	252	Low risk	Patients ≥ 18 years with CSDH with MGS 1–3	DXM for 20-day tapering course; starting with 16 mg/day for 4 days, reducing every 3 days	Burr hole craniostomy	Modified Rankin Scale at 3 months	DXM treatment was not found to be noninferior to burr-hole drainage with respect to functional outcomes and was associated with more complications and a greater likelihood of later surgery
S. Ng et al. [91]	HEMACORT; Corticosteroids as adjuvant treatment to surgery in CSDH	To compare the recurrence rate at 6 months in patients receiving post-operative prednisolone or placebo	2021	France	Multicenter, triple-blind, placebo-controlled RCT	155	Low risk	Patients ≥ 18 years with primary symptomatic CSDH (hypo- or isodense on imaging) treated with surgery	Prednisone within 72 h of surgery, 1 mg/kg/day for 1 week. Decreasing by 10 mg/week until 5 mg/day for last week (average 2 months)	Placebo as per prednisone regimen	Clinical and radiological recurrence of CSDH within 6 months postoperatively	Prednisone, as an adjuvant treatment to surgery, may reduce early radiological recurrence of CSDH, although clinical benefits are unclear
J. Tariq et al. [121]	Adjunctive postoperative DXM in CSDH	To compare the effect of BHC with and without a postoperative course of DXM on the recurrence rate of CSDH	2021	Pakistan	Single-center, parallel RCT	92	High risk	Patients ≥ 18 years with CSDH and required BHC	DXM initiated preoperatively (16 mg/day) and tapered over two weeks postoperatively following BHC reducing 3 mg every 3 days	BHC without DXM	Complication rate and recurrence of CSDH within 12 weeks postoperatively	Neurological and radiological outcome, and mortality rates were similar in both groups. The recurrence rate was lower and the complication rate higher in the DXM group, but not statistically significant
K. Mebberson et al. [75]	Adjuvant DXM with surgery for CSDH	To evaluate the efficacy and safety of adjuvant DXM in reducing recurrence rates after CSDH surgery with postoperative drainage	2020	Australia	Single-center, triple-blind, placebo-controlled RCT presenting interim results	47	High risk	Patients ≥ 18 years with symptomatic CSDH on CT requiring surgical evacuation	DXM for 14-day tapering course; starting with 16 mg/day for 3 days, reducing 4 mg every 3 days	Placebo as per DXM regimen	Postoperative mortality And recurrence rate requiring reoperation within 6 months, and postoperative morbidity by 30 days	Interim analysis suggested that DXM was safe and significantly reduced recurrence rates
**Tranexamic acid trials = 2**												
T. Yamada et al.* [133]	TXA and Goreisan preventing CSDH recurrence	To evaluate the efficacy of orally administered TXA and Goreisan in preventing recurrence after BHC for CSDH	2020	Japan	Single-center, 3-arm RCT	193	High risk	Patients undergoing initial BHC for CSDH	* TXA group: 750 mg orally per day from day after surgery for 3 months * Goreisan group: 7.5 g orally per day for 3 months	No medication	Recurrence of CSDH within 3 months requiring reoperation	Oral administration of TXA or Goreisan does not minimize recurrence after BHC for CSDH; however, TXA can reduce the haematoma volume
K. R. Wan et al. [128]	TXA for reducing CSDH recurrence	To assess whether adding TXA to standard neurosurgical treatment reduces reoperation rates for recurrent CSDH	2020	Singapore	Multicenter, open-label RCT	90	High risk	Patients ≥ 21 years with CSDH requiring surgery	Standard neurosurgical treatment with TXA (500 mg twice daily for 21 days)	Standard neurosurgical treatment only	Recurrence of CSDH requiring reoperation within 6 months postoperatively	The addition of TXA treatment did not significantly reduce recurrence. TXA did cause a delay in CSDH recurrence and a reduced residual haematoma volume at 6 weeks, but not sustained beyond 6 weeks
**Other pharmacological trials = 8**												
N. Fujisawa et al. [45]	Effect of Goreisan to prevent CSDH recurrence	To investigate whether Goreisan treatment decreases the recurrence rate of CSDH	2020	Japan	Single-center, parallel RCT	224	High risk	Patients who underwent initial surgery for symptomatic CSDH confirmed on CT	Goreisan (7.5 g three times a day for at least 3 months)	No medication	Symptomatic recurrence of CSDH within 3 months postoperatively	Adjuvant Goreisan treatment did not prove beneficial, although it approached significance to lower the recurrence rate in overall patients
T. Yamada et al.* [133]	TXA and Goreisan preventing CSDH recurrence	To evaluate the efficacy of orally administered TXA and Goreisan in preventing recurrence after BHC for CSDH	2020	Japan	Single-center, 3-arm RCT	193	High risk	Patients undergoing initial BHC for CSDH	* TXA group: 750 mg orally per day from day after surgery for 3 months * Goreisan group: 7.5 g orally per day for 3 months	No medication	Recurrence of CSDH within 3 months requiring reoperation	Oral administration of TXA or Goreisan does not minimize recurrence after BHC for CSDH; however, TXA can reduce the haematoma volume
Kamenova et al. [65]	SECA; CSDH and aspirin	To investigate the impact of continuing or discontinuing low-dose aspirin after CSDH surgery	2025	Switzerland	Double-blind, placebo-controlled RCT	150	Low risk	Patients ≥ 18 years with CSDH already taking low-dose aspirin (100 mg/day) for secondary prevention	Acetylsalicylic acid (ASA) (100 mg/day) for 12 days then resumed normal ASA treatment	Placebo for 12 days then resumed normal ASA treatment	Recurrence requiring reoperation within 6 months	Discontinuing ASA treatment did not reduce the recurrence rate of surgically treated CSDH within 6 months
M. Shafiei et al. [112]	Safety and timing of enoxaparin initiation after CSDH evacuation	To investigate the safety and timing of enoxaparin in the postoperative period in CSDH operated with BHC	2023	Iran	Multicenter, partially assessor-blinded RCT	136	High risk	Patients with CSDH eligible for surgical evacuation	Enoxaparin initiated 24 h after surgery	Enoxaparin initiated 72 h after surgery	Incidence of postoperative venous thromboembolism (VTE) and intracranial haemorrhagic events	In terms of VTE chemoprophylaxis, following BHC for CSDH, enoxaparin (early or late) will effectively prevent VTE development without any clinically significant rebleeding
H. Matsumoto et al. [73]	Japanese herbal medicine for preventing recurrent CSDH	To investigate the effect of 3 months of Goreisan versus Saireito for CSDH recurrence after surgical drainage	2024	Japan	Single-center, 3-arm RCT	118	High risk	Patients > 20 years who underwent initial surgery for symptomatic unilateral CSDH with objective neurological deficits	* Goreisan (6.0 g twice per day) for 3 months after surgery * Saireito (8.1 g twice per day) for 3 months after surgery	No medication	Symptomatic recurrence of CSDH requiring reoperation within 3 months postoperatively	Japanese herbal medicine (Goreisan and Saireito) significantly reduced postoperative recurrence in CSDH
D. Wang et al. [129]	CSDH treatment with ATO and low-dose DXM	To test the hypothesis that adding DXM to ATO potentiates the effects of ATO on CSDH	2020	China	Single-center, open-label, assessor-blinded, proof-of-concept RCT	60	High risk	Patients ≥ 18 years with primary CSDH with MLS < 1 cm, MGS < 3, and no immediate need for surgery	ATO (20 mg daily for 5 weeks) combined with low-dose DXM (2.25 mg daily for 2 weeks, 0.75 mg twice daily for 2 weeks, then once daily for 1 week)	ATO (20 mg daily for 5 weeks) alone	Haematoma reduction at 5 weeks	ATO + DXM resulted in significantly greater haematoma reduction and neurological improvement than ATO alone
K. Yang et al. [134]	Efficacy and safety of ATO for CSDH	To observe the effects of ATO on clinical efficacy, neurological function, inflammation, haematoma absorption, adverse reactions and recurrence rate in patients with CSDH	2020	China	Single-center, parallel RCT	58	High risk	Patients with symptomatic CSDH on imaging showing haematoma thickness < 5 mm, MLS < 1 cm, and suitable for conservative management	Oral ATO 20 mg daily for two months alongside standard conservative management	Standard conservative management with conventional nutritional neuropharmacology but without ATO	Effectiveness and safety of the treatment	Atorvastatin could reduce inflammatory response, promote haematoma absorption, protect neurological status and reduce the recrudescence rate for patients with CSDH
A. B. Pradhanang et al. [99]	Prophylactic phenytoin against postoperative seizure for CSDH patients	To evaluate the efficacy of prophylactic phenytoin in preventing early postoperative seizures in patients undergoing burr hole drainage for CSDH	2019	Nepal	Single-center parallel RCT	54	High risk	Patients ≥ 14 years with imaging confirmed CSDH requiring surgical intervention	Intravenous phenytoin (loading dose of 17 mg/kg followed by 100 mg three times daily for 7 days postoperatively)	No antiepileptic drug administration	Occurrence of early postoperative seizure	Incidence of postoperative seizure in patients undergoing burr hole drainage for CSDH was low. Routine prophylactic use of phenytoin, did not reduce seizure in the early postoperative period
**Surgical trials = 13**												
R. Raj et al. [100]	FINISH; Burr hole drainage with or without irrigation for CSDH	To test the therapeutic effect of haematoma irrigation	2024	Finland	Multicenter, double-blind (except operating neurosurgeon and OR staff), noninferiority RCT	589	Low risk	Patients ≥ 18 years with symptomatic CSDH requiring burr-hole drainage, hypo- or isodense on imaging	Burr hole drainage without irrigation	Burr hole drainage with irrigation with saline at body temperature (± 37 °C)	Symptomatic recurrence requiring reoperation within 6 months postoperatively	Burr hole drainage without irrigation was not found noninferior to drainage with irrigation and had a higher reoperation rate
A. Bartley et al. [11]	SIC!; Irrigation fluid temperature in the evacuation of CSDH	To investigate the effect of irrigation fluid temperature on CSDH recurrence rate	2022	Sweden	Multicenter RCT with blinding of patient, outcome assessor, and statistician	541	Low risk	Patients ≥ 18 years with CSDH requiring burr hole evacuation	Burr hole evacuation of CSDH with irrigation fluid at room temperature (± 22 °C)	Burr hole evacuation of CSDH with irrigation fluid at body temperature (± 37 °C)	Recurrence requiring reoperation within 6 months	Irrigation at body temperature was superior to irrigation at room temperature in terms of fewer recurrences
J. Duerinck et al. [33]	COMPACT; Optimal surgical treatment for CSDH	To evaluate the 30-day reoperation rate in CSDH for mini-craniotomy, twist drill craniostomy, and BHC	2022	Belgium	Multicenter 3-arm RCT	245	Some concerns	Patients ≥ 18 years with CSDH requiring surgery	* Mini-craniotomy (bone flap > 30 mm replaced and drain) * Twist drill craniostomy (twist drill burr hole < 5 mm and drain) * Burr hole craniostomy (2 burr holes 5–30 mm and drain)	None	Reoperation for recurrent or persistent CSDH within 30 days of primary surgery	All 3 techniques are effective at treating patients with CSDH with eventual 6-month outcome being similar. BHC offers the lowest recurrence rate combined with manageable complication rate (not statistically significant)
J. Soleman et al. [114]	cSDH-Drain-Trial; Subperiostal drainage (SPD) versus subdural drainage (SDD)	To investigate if SPD is noninferior to SDD in reducing recurrence rates of CSDH after burr-hole evacuation	2019	Switzerland	Multicenter, assessor-blinded, noninferiority RCT	220	Low risk	Patients ≥ 18 years with symptomatic CSDH on imaging requiring surgery	Subperiosteal drain	Subdural drain	Recurrence of CSDH indicating reoperation within 12 months	Although the noninferiority criteria were not met, SPD insertion led to lower recurrence rates, fewer surgical infections, and lower drain misplacement rates
D. Sale et al. [110]	Single versus double burr hole drainage for CSDH	To compare the recurrence rate following double burr holes and a single burr hole for CSDH	2021	Nigeria	Single-center, parallel RCT	192	High risk	Patients with imaging diagnosis of subacute or chronic SDH	Double burr hole drainage	Single burr hole drainage	Clinical recurrence within 6 months	A single burr hole is as efficacious as a double burr hole in terms of relief of symptoms and recurrence, and it has a shorter duration of surgery
Y. Bozhkov et al. [14]	Efficacy of subperiosteal drains in CSDH	To evaluate the efficacy of subperiosteal drain placement in reducing recurrence rates after BHC in patients with CSDH	2024	Germany	Single-center, parallel RCT	88	High risk	Patients ≥ 18 years with primary CSDH, suitable for BHC	Single BHC with saline irrigation followed by placement of a subperiosteal drain	Single BHC with saline irrigation without drain placement	Recurrence of CSDH requiring reoperation within 3 months postoperatively	Subperiosteal drain placement can be used safely and effectively to treat CSDH in conjunction with a single BHC procedure, significantly reducing the rate of recurrence
S. Zhang et al. [136]	A novel active bone hole drainage system for CSDH	To evaluate the efficacy of a novel active bone hole drainage system in reducing post-operative subdural pneumatosis (PSP) after CSDH surgery	2022	China	Single-center, assessor-blinded RCT	86	High risk	Patients with traumatic, symptomatic CSDH on imaging requiring surgery	Modified surgery with an active bone hole drainage system designed to reduce PSP by venting air during the procedure	Traditional drilling and drainage surgery without the additional drainage system	Incidence of PSP and primary endpoints (recurrence of CSDH and post-operative haematoma re-increasement) within 6 months	The modified surgery significantly reduced PSP incidence and primary endpoints compared to traditional surgery, improving prognosis
M. N. Stienen et al. [117]	CORRECT-SCAR; Covers to improve aesthetic outcome after surgery in CSDH	To evaluate if burr hole covers improve patient satisfaction of the scar in CSDH surgery	2024	Switzerland	Single-center, patient-blinded RCT	78	Low risk	Patients ≥ 18 years with primary CSDH (GCS > 8, able to communicate) requiring burr hole surgery	Any burr hole on intervention side is covered by a burr hole cover	None of the burr holes on the intervention side are covered by a burr hole cover	Patient satisfaction (using patient-reported outcome And scale 1–10) with the aesthetic result of the scar at 90 days	Satisfaction with the aesthetic result of the scar among CSDH patients after burr hole surgery is very high, and this study does not show evidence for improvement by applying a burr hole cover
T. Sun et al. [120]	Drilling drainage combined with intraoperative MMA occlusion for CSDH	To evaluate the therapeutic efficacy of drilling drainage combined with intraoperative MMA occlusion in managing CSDH	2024	China	Single-center, parallel RCT	72	Some concerns	Patients > 30 years with symptomatic CSDH on imaging requiring surgery, with MLS > 5 mm or haematoma thickness > 10 mm	Drilling drainage with intraoperative occlusion (severing) of the MMA using Digital Subtraction Angiography (DSA) guidance	Standard surgical drainage without MMA occlusion	Recurrence rate within 5 months postoperatively	MMA occlusion during drainage reduced the recurrence rate and shortened drainage tube retention time without increasing surgical complications
M. Mahmoodkhani et al. [71]	Half-saline (HS) vs. normal-saline (NS) in CSDH surgery	To evaluate the efficacy and safety of HS serum as an irrigation solution in CSDH surgery using BHC	2022	Iran	Multicenter, statistician-blinded RCT	61	High risk	Patients with CSDH eligible for BHC as treatment	HS irrigation during surgery	NS irrigation during surgery	Unspecified	HS as an irrigation fluid in BHC effectively reduced postoperative effusion and hospital stay duration without considerable complications
F. Roohollahi et al. [107]	Effect of number of burr holes on CSDH recurrence	To compare the effect of single burr hole versus double burr hole drainage on the recurrence rate of CSDH	2023	Iran	Single-center, assessor-blinded RCT	44	Low risk	Patients ≥ 18 years with primary CSDH on CT and with MLS > 5 mm requiring surgery	Single BHC with subperiosteal drainage and irrigation	Double BHC with subperiosteal drainage and irrigation	Recurrence rate of CSDH requiring reoperation within 6 months postoperatively	Single BHC was equally effective as double BHC in terms of recurrence rates but had a significantly shorter surgical duration. Single BHC is recommended for all patients
O. Akar et al. [7]	Simultaneous versus consecutive drainage of bilateral CSDH	To compare the clinical outcomes of simultaneous versus consecutive BHC for bilateral CSDH evacuation	2024	Turkey	Single-center, parallel RCT	43	Some concerns	Patients ≥ 18 years with symptomatic bilateral CSDH measuring > 10 mm in thickness on both sides	Simultaneous BHC of bilateral CSDH under general or local anesthesia with subdural drainage placement	Consecutive BHC, first evacuating the side with the thicker haematoma, followed by the contralateral side, with subdural drainage placement	Treatment success rate at 12 months, defined by absence of surgical complications, recurrence requiring reoperation, and good clinical outcomes	There were no significant differences in outcomes between the simultaneous and consecutive evacuation groups
K. Pathoumthong et al. [97]	Subdural drain (SDD) versus subperiosteal drain (SPD) for CSDH	To compare the clinical and radiographic outcomes of SDD versus SPD placement in patients undergoing burr hole evacuation for CSDH	2021	Thailand	Single-center parallel RCT	42	Some concerns	Patients ≥ 18 years with symptomatic CSDH on imaging	BHC followed by SDD placement (subdural drain inserted through frontal burr-hole and connected to a gravity drainage system)	BHC followed by SPD placement (subperiosteal drain connected to a suction drainage system)	Clinical And radiographic recurrence of CSDH within 6 months	Drain type (SDD or SPD) has no effect on the outcome. The surgeon’s preference determines which procedure is used
**Perioperative management trials = 8**												
T. S. R. Jensen et al. [60]	24vs48CSDH; Subdural drainage time after CSDH evacuation	To investigate the effect of drainage duration on recurrence and mortality after burr-hole evacuation of CSDH	2021	Denmark	Multicenter, double-blind (until 24 h postoperatively) RCT	420	Some concerns	Patients ≥ 60 years with symptomatic CSDH confirmed on imaging	48 h postoperative passive subdural drainage	24 h postoperative passive subdural drainage	Recurrence requiring reoperation within 90 days	No significant differences in the rates of recurrent haematoma or death during 90-day follow-up were identified between 24 and 48 h passive subdural drainage after burr hole evacuation of CSDH
M. Hjortdal Grønhøj et al. [49]	DRAIN TIME 2; Optimal drainage after evacuation of CSDH	To investigate the shortest possible drainage time without increasing the haematoma recurrence rate	2024	Denmark	Multicenter, assessor-blinded, 3-arm noninferiority RCT (patients and treating physicians blinded until 6 h post-surgery)	347	Some concerns	Patients ≥ 18 years with symptomatic CSDH, predominantly iso- or hypodense on imaging, undergoing single burr-hole evacuation with a passive subdural drain	* 6 h postoperative passive subdural drainage * 12 h postoperative passive subdural drainage	24 h postoperative passive subdural drainage	Symptomatic haematoma recurrence within 3 months requiring reoperation	6 h and 12 h drainage times resulted in significantly higher recurrence rates compared to 24 h. 24 h drainage is recommended as the standard approach
S. Sousa et al. [115]	GET-UP; Impact of early out-of-bed in postoperative outcomes of CSDH	To compare early mobilisation and 48 h bed rest on postoperative complications	2023	Portugal	Single-center, open-label RCT with retrospective outcome assessment review	208	Some concerns	Patients ≥ 18 years with CSDH requiring BHC drainage	Early mobilisation within 12 h post-surgery with closed subdural drains (removed 48 h post-surgery)	Bed rest with head of bed at 0° for 48 h and subdural drains removed > 48 h post-surgery	Medical complications until discharge: infections, venous thromboembolism, seizures	Early mobilization was associated with a reduction in medical complications without a significant effect on surgical recurrence, compared with a 48-h bed rest protocol
R. Hestin et al. [47]	NEURANESTH; General anaesthesia versus locoregional anaesthesia for CSDH	To compare the effect of local versus general anaesthetic during CSDH surgery on length of stay and functional outcome	2022	France	Single-center, assessor-blinded RCT	60	Low risk	Patients ≥ 18 years with CSDH requiring surgery	General anaesthesia with propofol (2.5 mg/kg), remifentanil (1 yg/kg), atracurium (0.5 mg/kg) and orotracheal intubation with mechanical ventilation	Locoregional anaesthesia with Lidocaine 1% for a scalp nerve block	Length of post-operative hospitalisation required until fit for following completion of medical checklist	In patients requiring CSDH evacuation, general and regional anaesthesia are comparable in terms of duration of time until medically fit for discharge and occurrence of postoperative complications
S. Anand et al. [8]	Superficial cervical plexus block for neck pain after CSDH surgery	To assess the effect of unilateral superficial cervical plexus block (SCPB) on managing neck pain as assessed by the numerical rating scale (NRS)	2024	India	Double-blind, placebo-controlled RCT	60	Low risk	Patients ≥ 18 years with unilateral CSDH, ASA grade I-III, undergoing BHC	Ultrasound-guided SCPB with 5 mL of 0.5% bupivacaine plus 1 mL of dexmedetomidine (50 µg/ml) administered preoperatively	Placebo SCPB using 5 ml of normal saline administered preoperatively	NRS pain score during neck movement in the postoperative period	Preoperative superficial cervical plexus block decreases neck pain and facilitates neck rotation in the postoperative period. It also decreases the analgesic requirement in the post-operative period
A. Kamel et al. [64]	Monitored anesthesia care of CSDH	To compare the efficacy and safety of combining dexmedetomidine with ketamine (DK) versus propofol (DP) for monitored anesthesia care during BHC for CSDH	2024	Egypt	Single-center RCT with blinding of patient and outcome assessor	56	Low risk	Patients 50–80 years, ASA physical status II-III, BMI 25–35 kg/m^2^, scheduled for burr hole evacuation of CSDH under monitored anesthesia care	DK group: Initial bolus of ketamine (1 mg/kg) and dexmedetomidine (1 µg/kg), followed by continuous infusion of ketamine (0.5 mg/kg/h) and dexmedetomidine (0.5 µg/kg/h)	DP group: Initial bolus of propofol (0.5 mg/kg) and dexmedetomidine (1 µg/kg), followed by continuous infusion of propofol (0.5 mg/kg/h) and dexmedetomidine (0.5 µg/kg/h)	Onset of sedation measured by the modified Observer’s Assessment of Alertness and Sedation score (OAA/S)	Combining dexmedetomidine with ketamine is safer than with propofol for effective monitored anesthesia care in high-risk patients undergoing BHC for CSDH, although it results in a longer onset of sedation
M. M. Mohamed et al. [80]	Magnesium sulphate adjuvant during scalp block for CSDH surgery	To evaluate the efficacy and safety of magnesium sulphate as an adjuvant to bupivacaine and lidocaine during scalp block in patients undergoing CSDH evacuation surgery	2024	Egypt	Single-center RCT with blinding of patient and investigator	38	Low risk	Patients ≥ 45 years with unilateral CSDH, ASA physical status II or III, and GCS 14–15 undergoing surgical evacuation	Scalp block with lidocaine (1%) and bupivacaine (0.25%) combined with 5 mL of magnesium sulphate (500 mg)	Scalp block with lidocaine (1%) and bupivacaine (0.25%) without magnesium sulphate	Time to first dose of postoperative rescue analgesia	Magnesium sulphate can effectively and safely be used as an adjuvant to bupivacaine and lidocaine during scalp block for postoperative pain management
R. Fahmy et al. [41]	Magnesium sulphate versus fentanyl sedation during BHC	To compare the hemodynamic and analgesic effects of magnesium sulphate versus fentanyl as adjuncts to propofol-induced conscious sedation in CSDH evacuation	2022	Egypt	Single-center, triple-blind RCT	32	Low risk	Patients > 50 years with unilateral CSDH, ASA physical status I–II, And GCS 14–15, requiring BHC	Magnesium sulphate infusion (loading dose of 50 mg/kg followed by continuous infusion at 15 mg/kg/h) combined with propofol sedation	Fentanyl infusion (loading dose of 1 μg/kg followed by continuous infusion at 0.5 μg/kg/h) combined with propofol sedation	Average intraoperative systolic blood pressure	Magnesium sulphate resulted in better hemodynamic stability and less incidence of nausea and vomiting compared to fentanyl
**MMAE trials = 5**												
J. Liu et al. [70]	MAGIC-MT; Usual care versus MMAE	To determine whether MMA embolisation reduces the risk of symptomatic recurrence or progression of nonacute SDH	2024	China	Multicenter RCT with partial PROBE design	722	High risk	Patients ≥ 18 years with symptomatic nonacute SDH on imaging, producing mass effect, and with a premorbid modified Rankin Scale of 0–2	MMA embolisation using the Onyx liquid embolic system, performed adjunctively to surgical or nonsurgical standard treatment	Usual care, including burr hole drainage or conservative management as decided by the treating physician	Symptomatic recurrence or progression of SDH within 90 days	MMA embolisation did not significantly reduce recurrence or progression at 90 days compared to usual care but did reduce serious adverse events
J. Davies et al. [29]	EMBOLISE; Adjunctive MMAE for CSDH	To assess whether MMA embolisation adjunct to surgery reduces the risk of haematoma recurrence or progression in CSDH patients	2024	USA	Multicenter, partially assessor-blinded, adaptive-design RCT	400	High risk	Patients 18–90 years with symptomatic subacute or chronic SDH on CT requiring surgical evacuation, with haematoma thickness > 15 mm or MLS ≥ 5 mm	Surgical evacuation combined with MMA embolisation using the Onyx liquid embolic system	Surgical evacuation alone	Haematoma recurrence or progression leading to reoperation within 90 days	MMA embolisation significantly reduced the risk of haematoma recurrence or progression requiring reoperation. Further study is needed to evaluate the safety of MMA embolisation
E. Shotar et al. [113]	EMPROTECT; MMAE to prevent CSDH recurrence	To investigate whether MMA embolisation after surgery reduces the risk of CSDH recurrence in patients at high risk of postoperative recurrence	2025	France	Multicenter RCT with PROBE design	342	Low risk	Patients ≥ 18 years who have undergone BHC for a recurrent CSDH or for a primary CSDH with at least one high-risk factor for recurrence	MMA embolisation with microparticles within 7 days after BHC	Standard medical care	Recurrence of CSDH within 6 months	MMA embolisation did not lead to a significantly lower rate of recurrence at 6 months compared with standard medical care alone
D. Fiorella et al. [43]	STEM; Adjunctive MMAE versus standard treatment	To evaluate whether adjunctive MMA embolisation reduces the risk of treatment failure in patients with symptomatic CSDH	2024	USA	Multicenter, open-label, international RCT	310	High risk	Patients ≥ 18 years with symptomatic CSDH on imaging (≥ 50% iso- or hypodense) measuring > 10 mm in thickness	MMA embolisation using the Squid liquid embolic system, performed adjunctively to surgical or nonsurgical standard treatment	Standard treatment alone, either surgical or nonsurgical as decided prior to randomization	Composite of recurrent or residual CSDH (> 10 mm), reoperation or surgical rescue, or major disabling stroke, myocardial infarction, or death from neurologic causes at 180 days	Adjunctive MMA embolisation significantly reduced the risk of treatment failure without increasing the incidence of disabling stroke or death. The benefit was greater in patients receiving nonsurgical standard treatment
S. Ng et al. [92]	MMAE as adjuvant to surgery for CSDH	To assess the effect of MMA embolisation as an adjuvant to surgery on haematoma volume resorption in symptomatic CSDH patients	2020	France	Single-center, assessor-blinded, pilot RCT	46	Some concerns	Patients ≥ 18 years with symptomatic primary CSDH on CT requiring surgery	Surgical treatment (twist drill craniostomy or craniotomy with drainage) followed by MMA embolisation	Surgical treatment alone (twist drill craniostomy or craniotomy with drainage)	Haematoma volume resorption at 3 months postoperatively	MMA embolisation significantly improved haematoma volume resorption at 3 months. Both groups had one recurrence, with no embolisation-related complications
**Miscellaneous trials = 1**												
K. Osuka et al. [94]	Sequential expression of chemokines in CSDH fluids after surgery	To analyze the sequential expression of chemokines in CSDH fluids after trepanation surgery with or without irrigation	2021	Japan	Single-center, parallel RCT	20	Some concerns	Patients undergoing trepanation surgery for CSDH	Irrigation of CSDH fluid with artificial cerebrospinal fluid during surgery, followed by drainage	Non-irrigation, with placement of a drainage tube without fluid irrigation	Changes in chemokine concentrations in CSDH fluids over time	Neutrophil- and macrophage-attracting chemokines increased by day 1 post-surgery, regardless of irrigation, suggesting their key role in early healing

*Trials addressing multiple topics are represented in multiple entries within the table

*ASA* American Society of Anesthesiologists, *ATO* atorvastatin, *BHC* burr hole craniostomy, *CSDH* chronic subdural haematoma, *CT* computed tomography, *DXM* dexamethasone, *GCS* Glasgow Coma Scale, *IV* intravenous, *MGS* Markwalder Grading Scale, *MLS* midline shift, *OR* operating room, *PROBE* Prospective Randomised Open, Blinded Endpoint, *RCT* randomised controlled trial, *RoB* risk of bias, *TXA* tranexamic acid

**Table 3:** Overview of all currently running RCTs in CSDH included in review

**Trial name; title** *Registered number*	**Country**	**Primary objective**	**Trial design**	**Completion date**	**N**	**Patients**	**Intervention**	**Comparison**	**Outcome (primary)**
**Steroid trials = 3**									
ATOCH II; Atorvastatin combined with dexamethasone in CSDH* ChiCTR1900021659 [17]	China	To evaluate the clinical efficacy and safety of atorvastatin combined with dexamethasone for CSDH patients	Multicenter, double-blind, placebo-controlled RCT	Unknown	240	Patients 18–90 years with CSDH on imaging and mRS 1–3, requiring surgical treatment	ATO 20 mg daily + DXM (0.75 mg thrice per day for 2 weeks, twice per day for 1 week, once per day for 1 week) for 4 weeks in total	ATO 20 mg daily + placebo as per DXM regimen for 4 weeks in total	Comprehensive outcome within 4 weeks
ADTCSDH; Combining atorvastatin and dexamethasone for CSDH after surgery* ChiCTR2000030156 [18]	China	To explore the clinical effect of combining atorvastatin and dexamethasone in the treatment of CSDH drilling and drainage	Multicenter, 3-arm, open-label RCT	Unknown	90	Patients 18–90 years with symptomatic CSDH on imaging undergoing surgical drilling and drainage	* ATO 20 mg daily for 4 weeks * ATO 20 mg daily + DXM (0.75 mg thrice per day for 2 weeks, twice per day for 1 week, once per day for 1 week) for 4 weeks in total	Standard postoperative neurosurgical monitoring	Recurrence rate within 12 weeks
ADELE; ATO and DXM in CSDH* RPCEC00000436 [108]	Cuba	To evaluate the efficacy and safety of ATO and DXM in the postoperative treatment of patients with CSDH	Open-label, 4-arm parallel RCT	April 2026	48	Patients 19–80 years with CSDH on imaging requiring surgical treatment	* ATO and conventional treatment (ATO for 8 weeks: 20 mg/day) * DXM and conventional treatment (DXM for 4 weeks: 16 mg IV days 1–3, reducing every 3 days to 4 mg orally daily from day 11) * ATO, DXM, and conventional treatment	Conventional treatment (supportive treatment) during hospital admission	Reduction in volume and reduction in recurrences at baseline, the 7th day and at a month
**Tranexamic acid trials = 7**									
TORCH; Tranexamic acid to prevent operation in CSDH NCT03582293 [125]	Netherlands	To investigate the efficacy and safety of TXA as primary conservative treatment of CSDH	Multicenter, double-blind, placebo-controlled RCT	October 2029	554	Patients ≥ 50 years with CSDH on CT safe for primary conservative treatment based on symptoms and signs	TXA 1 g/day for 28 days	Placebo as per TXA regimen	Surgery for CSDH within 12 weeks of start of treatment
TXA for treatment of CSDH NCT06718751 [124]	USA	To establish the safety and efficacy of using TXA to resolve CSDH without the need for surgical intervention	Double-blind, placebo-controlled RCT	January 2026	300	Patients 21–95 years with CSDH and eligible for conservative treatment	TXA 650 mg/day for 21 weeks	Placebo as per TXA regimen	Rate of resolution measured as CSDH volume or diameter at 6, 12, And 21 weeks
TABASCO; TXA versus MMA embolisation as an adjunctive therapy* DRKS00033515 [31]	Germany	To determine whether postoperative treatment of CSDH using adjuvant TXA is equivalent to MMA embolisation	Multicenter, open-label RCT	Unknown	276	Patients ≥ 18 years who underwent surgery for primary unilateral CSDH < 24 h before inclusion	Postoperative adjuvant drug therapy with TXA	Postoperative adjuvant MMA embolisation on the affected side using digital subtraction angiography	Postoperative volume decrease through time within 3 months
TRACS: TXA in CSDH NCT02568124 [82]	Canada	To investigate if TXA can be used as conservative treatment for CSDH, lowering surgical procedures and decreasing recurrence	Multicenter, double-blind, placebo-controlled RCT	June 2025	130	Patients ≥ 18 years with CSDH confirmed on CT containing a chronic component	TXA 750 mg/day until complete radiological resolution of CSDH or a maximum of 20 weeks	Placebo daily until complete radiological resolution of CSDH or a maximum of 20 weeks	Rate of CSDH resolution at 20 weeks without intervening unplanned surgical procedure (surgery is at discretion of treating team)
TRACE 2; TXA in the treatment of residual CSDH NCT05713630 [122]	Canada	To evaluate the effectiveness of TXA in improving patient-reported quality of life and reducing CSDH recurrence	Multicenter, triple-blind RCT	November 2030	130	Patients ≥ 45 years with symptomatic CSDH ≥ 8 mm in thickness on CT (any density), requiring either conservative management or surgery	Standard care + TXA: 1 g oral or IV loading dose followed by 500 mg TXA orally or IV three times daily for 45 days	Standard care + placebo: identical capsule or IV saline injection, mimicking the TXA regimen	European Quality of Life 5 Dimensions 5 Level (EQ-5D-5L) questionnaire every two weeks for 45 days
TRACE; TXA in the treatment of residual CSDH NCT04898712 [123]	Canada	To compare haematoma volume 4–8 weeks after CSDH surgery in patients receiving TXA versus no treatment	Double-blind, placebo-controlled RCT	Recruitment complete December 2023	90	Patients ≥ 18 years with CSDH requiring surgery	TXA for 4–8 weeks post-surgery: < 60 kg: 1 g/day 60–100 kg: 1.5 g/day > 100 kg: 2 g/day	Placebo as per TXA regimen	Change in haematoma volume in mL from immediately post-operative to repeat CT at 4–8 weeks
Postoperative TXA in CSDH patients Not registered [30]	Brazil	To determine whether postoperative TXA reduces risk of complications and CSDH recurrence after BHC	Single-center, open-label RCT	December 2025	Unknown (50 in pilot)	Patients ≥ 18 years with symptomatic CSDH undergoing BHC	Postoperative TXA at 750 mg/day in 3 daily doses for 3 months	No TXA	Recurrence requiring reoperation within 6 months
**Other pharmacological trials = 8**									
REACH; Atorvastatin in CSDH NCT03956368 [101]	Hong Kong	To evaluate the efficacy and safety of atorvastatin in patients with CSDH	Multicenter, double-blind, placebo-controlled RCT	Recruitment complete March 2023	690	Patients ≥ 18 years with CSDH on CT	ATO 20 mg daily for 8 weeks	Placebo as per ATO regimen	Favourable mRS score of 0–3 at 6 months
Peiyuan Huayu formula for CSDH treatment ChiCTR2300072255 [23]	China	To develop a new plan for the treatment of CSDH by combining traditional Chinese and Western medicine	Multicenter, statistician-blinded RCT	December 2025	400	Patients 18–85 years with CSDH on imaging	Conventional treatment and Peiyuan Huayu formula	Conventional treatment	Haematoma volume and recurrence time of CSDH
RELACS; Restarting early versus later anticoagulation for CSDH with atrial fibrillation NCT06696079 [105]	Finland	To assess the benefit of early resumption versus late resumption of oral anticoagulation medication in adults with atrial fibrillation undergoing surgery for CSDH	International, multicenter, assessor-blinded RCT	June 2027	332	Patients ≥ 18 years with symptomatic CSDH (predominantly hypo- or isodense on imaging) requiring surgery and previously prescribed oral anticoagulation medication due to atrial fibrillation	Early resumption: oral anticoagulation therapy is resumed on the 5th postoperative day	Late resumption: oral anticoagulation therapy is resumed on the 30th postoperative day	Composite outcome: thromboembolic events, hemorrhagic events, and vascular death within 90 days
ATOCH II; Atorvastatin combined with dexamethasone in CSDH* ChiCTR1900021659 [17]	China	To evaluate the clinical efficacy and safety of atorvastatin combined with dexamethasone for CSDH patients	Multicenter, double-blind, placebo-controlled RCT	Unknown	240	Patients 18–90 years with CSDH on imaging and mRS 1–3, requiring surgical treatment	ATO 20 mg daily + DXM (0.75 mg thrice per day for 2 weeks, twice per day for 1 week, once per day for 1 week) for 4 weeks in total	ATO 20 mg daily + placebo as per DXM regimen for 4 weeks in total	Comprehensive outcome within 4 weeks
CHARM; Chinese herbal formula HuoXue LiShui for CSDH NCT06427980 [38]	China	To compare haematoma progression or recurrence requiring reoperation at 24 weeks in patients with CSDH treated with HuoXue LiShui and placebo	Multicenter, double-blind, placebo-controlled RCT	March 2026	160	Patients 18–90 years with primary CSDH or residual haematoma after burr hole drainage, with no immediate need for surgery (GCS ≥ 14 and mRS ≤ 2)	Chinese Herbal formula HuoXue LiShui, one bag (28.5 g) twice daily after meals for 8 weeks	Placebo as per HuoXue LiShui regimen	Rate of (re)operation within 24 weeks
ADTCSDH; Combining atorvastatin and dexamethasone for CSDH after surgery* ChiCTR2000030156 [18]	China	To explore the clinical effect of combining atorvastatin and dexamethasone in the treatment of CSDH drilling and drainage	Multicenter, 3-arm, open-label RCT	Unknown	90	Patients 18–90 years with symptomatic CSDH on imaging undergoing surgical drilling and drainage	* ATO 20 mg daily for 4 weeks * ATO 20 mg daily + DXM (0.75 mg thrice per day for 2 weeks, twice per day for 1 week, once per day for 1 week) for 4 weeks in total	Standard postoperative neurosurgical monitoring	Recurrence rate within 12 weeks
ADELE; ATO and DXM in CSDH* RPCEC00000436 [108]	Cuba	To evaluate the efficacy and safety of ATO and DXM in the postoperative treatment of patients with CSDH	Open-label, 4-arm parallel RCT	April 2026	48	Patients 19–80 years with CSDH on imaging requiring surgical treatment	* ATO and conventional treatment (ATO for 8 weeks: 20 mg/day) * DXM and conventional treatment (DXM for 4 weeks: 16 mg IV days 1–3, reducing every 3 days to 4 mg orally daily from day 11) * ATO, DXM, and conventional treatment	Conventional treatment (supportive treatment) during hospital admission	Reduction in volume and reduction in recurrences at baseline, the 7th day and at a month
Tissue plasminogen activator (tPA) for clearing CSDH NCT05491356 [86]	Canada	To determine the utility of tPA, administered intra-catheter during twist drill craniostomy, in the clearance of CSDH	Triple-blind, placebo-controlled pilot RCT	Recruitment complete July 2024	40	Patients ≥ 18 years with symptomatic CSDH requiring surgical drainage via twist drill craniostomy	Intra-catheter administration of 2 mL tPA during twist drill craniostomy	Intra-catheter administration of 2 mL sterile 0.9% saline solution during twist drill craniostomy	Study feasibility, assessed by patient recruitment rate, protocol adherence, and unexpected events within 8 months
**Surgical trials = 10**									
CSDH removal with or without intraoperative irrigation jRCT1041220124 [62]	Japan	To evaluate the effectiveness of irrigation subsequent to the evacuation of CSDH utilizing artificial spinal fluid	Open-label, parallel RCT	February 2026	1186	Patients ≥ 20 years old with symptomatic CSDH requiring surgical intervention of a single side	BHC followed by irrigation with ≥ 200 mL of artificial spinal fluid until the fluid is clear. A closed drain remains for ≥ 12 h postoperatively	BHC without irrigation, with drain placement alone and a closed drain for at least 12 h postoperatively	Reoperation within 6 months postoperatively
SUPERDURA; Active subperiosteal versus passive subdural drainage NCT06621407 [2]	Denmark	To test the hypothesis that 24 h active subperiosteal drainage is noninferior to 24 h passive subdural drainage after single burr hole evacuation of a unilateral CSDH	Multicenter, noninferiority RCT with blinding of patient and outcome assessor	April 2028	598	Patients aged ≥ 18 years with unilateral CSDH on imaging undergoing single burr hole evacuation	24 h active subperiosteal drainage	24 h passive subdural drainage	Composite outcome: mortality and recurrent CSDH requiring reoperation within 90 days
Different skull burr hole sites and CSDH prognosis ChiCTR2000033967 [19]	China	To compare the efficacy of different skull bore hole sites on the prognosis of CSDH	2-arm parallel RCT	Recruitment complete December 2022	200	Patients with CSDH requiring surgical treatment	Burr hole via frontal bone	Burr hole via parietal bone	Haematoma residual volume and ratio, gas volume in haematoma cavity, recurrence, modified MGS and mRS score
Intraoperative irrigation in CSDH JPRN-UMIN000040527 [126]	Japan	To study the benefit of adding irrigation to simple drainage in CSDH surgery	2-arm, parallel RCT	Unknown	120	Patients ≥ 20 years with novel CSDH requiring surgery	Burr hole drainage with irrigation	Burr hole drainage	Recurrence rate
Augmented reality for subdural drain placement NCT06052124 [87]	USA	To assess the accuracy and feasibility of augmented reality (AR) guided subdural evacuating portal sytem (SEPS) placement	Single-center, open-label RCT with sequential assignment	December 2025	100	Patients 18–90 years with chronic or subacute SDH planned to be treated by SEPS	AR-guided SEPS placement (first as a pilot for usability in five patients, then to guide SEPS drains)	Standard anatomical guided SEPS placement	Accuracy of SEPS placement, measured in mm difference between AR-guided and standard placement based on post-procedure CT scans
Subperiosteal versus subdural drain after BHC CTRI/2024/10/075273 [28]	India	To find if the recurrence rate of CSDH when treated with subperiosteal drainage is noninferior to subdural drain placement at three months	Noninferiority RCT with blinding of patient and outcome assessor	November 2026	100	Patients ≥ 18 years with symptomatic CSDH on imaging or asymptomatic CSDH with midline shift > 10 mm requiring surgery	Subperiosteal placement of the drainage catheter	Subdural placement of the drainage catheter	Recurrence rate requiring reoperation within 3 months
Minimally invasive puncture and burr hole drainage in CSDH patients ChiCTR2200059808 [22]	China	To compare the clinical efficacy of minimally invasive puncture and drilling drainage in the treatment of CSDH	2-arm, parallel RCT	Unknown	80	Patients ≥ 18 years with symptomatic CSDH (predominantly hypo- or isodense on imaging) requiring burr hole evacuation	Minimally invasive puncture	Burr hole drainage	Recurrence rate, mortality, cure rate, and surgical failure rate
Endoscopic evacuation versus craniotomy for evacuating CSDH CTRI/2023/08/057000 [27]	India	To compare the effectiveness of endoscopic evacuation versus craniotomy for evacuating of CSDH	Single center, open-label RCT	September 2025	60	Patients with CSDH requiring surgical intervention	Endoscopic evacuation via a single burr hole	Craniotomy and evacuation of CSDH	Recurrence rate at 1, 3, and 6 months after surgery
Evaluation of siphon method in CSDH IRCT20211130053232N1 [57]	Iran	To compare the possible advantages and disadvantages of siphon washing method with traditional method in CSDH	2-arm, parallel RCT with blinding of patient and outcome assessor	Unknown	60	Patients with CSDH on CT with neurological deficit, GCS decline, and/or gait disorder	Burr hole drainage utilizing the siphon method	Standard burr hole drainage	Recurrence rate within 12 months
Neuroendoscopic and non-endoscopic surgical techniques in treating septated CSDH IRCT20190618043934N14 [56]	Iran	To compare neuroendoscopic and non-endoscopic surgical techniques in treating septated CSDH	Single-center RCT with blinding of patient and outcome assessor	Recruitment complete September 2022	60	Patients diagnosed with mixed-density CSDH on imaging	Neuroendoscopic surgery	Standard surgical management	Duration of surgery, mortality, morbidity, recurrence rate within 6 months, and duration of hospitalization
**Perioperative management trials = 7**									
BP-CSDH; Body posture in preventing CSDH Recurrence NCT06401772 [36]	China	To investigate the effectiveness and safety of body posture to improve intracranial pressure in CSDH	Multicenter, assessor-blinded RCT	January 2028	830	Patients ≥ 60 years with symptomatic CSDH, with thickness ≥ 10 mm and MGS ≤ 2, undergoing burr hole drainage	Intracranial hypotension targeted body posture, requiring lower limb elevation (30°) over head level for 3 months post-surgery when possible	Standard supine positioning post-surgery	Symptomatic recurrence with haematoma thickness ≥ 10 mm within 90 days postoperatively
ECHO; Exhaustive versus fixed-time drainage for CSDH NCT04573387 [103]	China	To evaluate whether exhaustive drainage after one burr-hole craniostomy reduces recurrence rates and improves clinical outcomes	Multicenter, assessor-blinded RCT	Recruitment complete December 2024	309	Patients 18–90 years with symptomatic CSDH on imaging	Exhaustive drainage following BHC, where the drain remains until haematoma volume is minimized, with up to 3 urokinase injections if needed	Fixed-time drainage following BHC, where the drain is removed 48 h postoperatively	Recurrence requiring reoperation within 6 months postoperatively
Nerve block and sedative anesthesia versus general anesthesia NCT05888389 [109]	China	To evaluate the safety of nerve block anesthesia combined with sedative anesthesia versus general anesthesia during BHC with drainage for CSDH	Multicenter, assessor-blinded, noninferiority RCT	December 2025	190	Patients 18–80 years with CSDH requiring surgical treatment and with haematoma thickness > 10 mm or midline shift > 10 mm	Cranial nerve block anesthesia combined with IV dexmedetomidine for sedation at a rate of 2−4ug/kg for 10 min, followed by a continuous infusion of 0.5-1ug/kg/h until RASS −3	General anesthesia with propofol or etomidate, sufentanil, rocuronium or cisatracurium	Incidence of intraoperative body movement
Dexmedetomidine during awake craniotomy PACTR202408593043200 [95]	Egypt	To detect role of administration of dexmedetomidine during awake craniotomy on the inflammatory and stress response, potentially improving postoperative recovery	Patient-blinded, parallel RCT	Recruitment complete December 2024	62	Patients 18–90 years with CSDH and scheduled for elective craniotomy	Awake craniotomy with dexmedetomidine: loading dose of dexmedetomidine at 1 μg/kg over 10 min, followed by a continuous infusion of 0.2–0.7 μg/kg/hr during the surgery	General anesthesia with agents such as propofol, fentanyl, and sevoflurane	Levels of inflammatory markers (C-reactive protein [CRP], interleukin-6 [IL-6], interleukin-8 [IL-8]) measured preoperatively, immediately postoperatively, and at 24 and 48 h postoperatively
Ketofol versus dexmedetomidine as sedation for CSDH surgery CTRI/2022/09/045494 [26]	India	To compare dexmedetomidine to ketofol for sedation to keep patients hemodynamically stable during operation	Single-center, open-label RCT	Recruitment complete September 2023	60	Patients 20–65 years with CSDH requiring burr hole evacuation	Dexmedetomidine 1 µg per kg as loading dose followed by infusion at 0.2–0.7 mcg/kg/hr IV	Ketofol 0.5 mg per kg per hour IV	Intraoperative hemodynamics
ABC-SDH trial I; Awake BHC in CSDH patients DRKS00034040 [32]	Germany	To determine whether awake burr hole trepanation under local anesthesia is a safe and feasible alternative to general anesthesia	Single-center, open-label, safety and feasibility RCT	Recruitment complete December 2024	50	Patients ≥ 18 years with CSDH on imaging requiring surgery	Burr hole trepanation performed under local anesthesia (awake surgery)	Burr hole trepanation performed under general anesthesia	Recruitment rate (consent rate and intended surgery rate after randomization) and complication rate within 1 month postoperatively
HOPE; High concentration oxygen for pneumocephalus NCT04725851 [48]	Hong Kong	To determine whether high-concentration oxygen therapy reduces pneumocephalus volume after burr hole drainage for CSDH	Single-center, assessor-blinded RCT	Recruitment complete December 2024	36	Patients ≥ 18 years with CSDH on imaging, treated with BHC, and presenting with postoperative pneumocephalus	High-concentration oxygen therapy 12–15 L/min via Non-Rebreather Mask for 24 h	Room air or low-concentration oxygen therapy (0–2 L/min via nasal cannula)	Reduction in pneumocephalus volume after 24 h on CT
**MMAE trials = 21**									
PREMMA; Puerto Rico MMAE for CSDH trial NCT06466733 [89]	Puerto Rico	To compare the effectiveness of MMA embolisation as a standalone treatment versus standard surgical evacuation via burr hole or craniotomy	Multicenter, assessor-blinded RCT	July 2031	658	Patients ≥ 21 years, with CSDH on imaging requiring treatment, with GCS ≥ 14 and MGS ≤ 2	MMA embolisation	Standard surgical evacuation, either BHC or craniotomy	Recurrence requiring reoperation at 3, 6, and 12 months
COMPLEMENT; MMAE for CSDH patients NCT06772740 [102]	Japan	To assess the efficacy and safety of MMA embolisation for CSDH	2-arm parallel RCT with PROBE design	March 2029	600	CSDH patients ≥ 18 years with pre-mRS 0–3, haematoma thickness ≥ 10 mm, and ≥ 1 risk factor (e.g. age ≥ 75 years, bilateral haematoma, etc.)	Conventional treatment with MMA embolisation within 7 days after randomization	Conventional treatment	Rate of recurrence of CSDH within 6 months
LEADH; Cyanocrylate embolisation adjuvant to standard of care NCT05374681 [68]	France	To evaluate the effectiveness of MMA embolisation in CSDH	Multicenter, assessor-blinded RCT	September 2026	550	Patients ≥ 18 years with CSDH > 10 mm on CT, localized to convexity	MMA embolisation with cyanoacrylates adjunct to surgical or conservative management as decided by the neurosurgeon	Surgical or conservative management alone as decided by the neurosurgeon	Clinical or radiographic recurrence of CSDH within 6 months
CHESS; CSDH with embolisation versus surgery NCT06347796 [88]	USA	To test in moderately symptomatic CSDH patients if MMA embolisation can be used as an alternative to conventional open surgery	Multicenter, assessor-blinded RCT	September 2028	520	Patients 40–90 years with unilateral CSDH (≥ 10 mm in thickness) or bilateral CSDH (one side no symptoms and < 5 mm in thickness). CSDH at least 2/3 iso- or hypodense	Particle MMA embolisation	Surgical drainage through BHC or craniotomy	Need for reoperation or death within 180–210 days
METRICS; MMAE for high-risk intractable CSDH ChiCTR2000032464 [21]	China	To evaluate whether MMA embolisation reduces treatment failure compared to traditional treatment strategies in patients with high risk for CSDH recurrence	Multicenter, assessor-blinded RCT	Recruitment complete December 2024	516	Patients ≥ 18 years with CSDH on imaging and at least one risk factor for recurrence (e.g. > 80 years, use of antithrombotics, bilateral CSDH)	MMA embolisation, either alone (for non-surgical patients) or adjunct to surgical evacuation (for surgical patients)	Traditional strategies, including oral atorvastatin (20 mg daily for 8 weeks) for non-surgical patients and BHC for surgical patients	Treatment failure defined as substantial residual haematoma, haematoma enlargement, or recurrence/haematoma progression requiring reoperation
Endovascular Therapy Combined with Operation vs Simple operation ChiCTR2000039359 [20]	China	To compare the prognosis of burr hole drainage standalone or combined with MMA embolisation in the treatment of CSDH	2-arm, parallel RCT	Unknown	480	Patients 18–70 years with symptomatic CSDH on imaging (hypo- or isodense, or mixed density with acute component < 50%) in need of surgical treatment	MMA embolisation combined with burr hole drainage	Burr hole drainage	Recurrence rate
OTEMACS; Onyx™ Trial for MMAE for CSDH NCT04742920 [84]	France	To assess the safety and effect on recurrence rate and functional outcome of endovascular treatment in patients with CSDH	Multicenter, RCT with PROBE design	September 2025	440	Patients ≥ 18 years with symptomatic CSDH on imaging and pre-mRS ≤ 3	MMA embolisation in addition to standard management with Onyx non-adhesive liquid embolic agent	Standard management (surgical or conservative)	Recurrence rate within 90 days
MMAE versus surgery for CSDH ChiCTR2500096266 [16]	China	To evaluate the safety and efficacy of MMA embolisation compared with surgery in the treatment of CSDH	Multicenter, assessor-blinded RCT	October 2027	386	Patients 18–90 years with symptomatic CSDH (< 30% high-density components) and haematoma thickness > 15 mm or MLS > 5 mm	MMA embolisation + standard drug adjuvant therapy	Surgery + standard drug adjuvant therapy	Recurrence rate within 90 days
MEMBRANE; MMAE for CSDH with TRUFILL n-BCA NCT04816591 [78]	USA	To investigate the effect of MMA embolisation as an adjunct to standard of care treatment for CSDH	Multicenter, open-label RCT	May 2025	376	Patients 18–90 years with confirmed CSDH and a pre-randomization mRS ≤ 3	MMA embolisation using TRUFILL n-BCA, in addition to standard surgical or medical management	Standard of care surgery or medical management only	Recurrence or progression of CSDH requiring re-intervention within 180 days
Embotrial-1; Endovascular vs conservative CSDH treatment NCT06274580 [39]	Italy	To assess the superiority of MMA embolisation over conservative treatment	Multicenter RCT with PROBE design	January 2028	300	Patients ≥ 18 years with CSDH with MGS ≤ 1, haematoma width ≤ 20 mm, MLS ≤ 7 mm, and pre-mRS score ≤ 2	MMA embolisation using polyvinyl alcohol particles or liquid embolizing materials	Standard conservative management	Rate of incomplete haematoma resolution or need for surgical rescue within 6 months
SWEMMA; Swedish trial on MMAE versus surgery NCT05267184 [85]	Sweden	To compare efficacy of MMA embolisation on rates of reoperation in CSDH patients with mild to moderate symptoms	Multicenter, asessor-blinded RCT	March 2027	288	Patients 18–89 years with CSDH requiring surgical treatment, MGS < 2, and GCS > 13	MMA embolisation with liquid embolic agents	Surgical drainage through BHC or craniotomy	Reoperation rate of CSDH within 3 months
TABASCO; TXA versus MMAE as an adjunctive therapy* DRKS00033515 [31]	Germany	To determine whether postoperative treatment of CSDH using adjuvant TXA is equivalent to MMA embolisation	Multicenter, open-label RCT	Unknown	276	Patients ≥ 18 years who underwent surgery for primary unilateral CSDH < 24 h before inclusion	Postoperative adjuvant drug therapy with TXA	Postoperative adjuvant MMA embolisation on the affected side using digital subtraction angiography	Postoperative volume decrease through time within 3 months
EMMA-Can; Management of CSDH with or without EMMA NCT04750200 [72]	Canada	To investigate the effect of MMA embolisation as an adjunct to standard of care treatment for minimizing CSDH recurrence	Open-label parallel RCT	March 2026	200	Patients ≥ 18 years with baseline mRS ≤ 2 and with symptomatic CSDH requiring surgical drainage or haematoma thickness ≥ 10 mm	MMA embolisation as an adjunct to the standard of care	Standard of care, including medical and/or surigcal treatment	Radiographic recurrence of CSDH within 90 days
STORMM; MMAE for CSDH NCT06163547 [77]	Switzerland	To evaluate the efficacy of MMA embolisation in CSDH	Multicenter, open-label RCT	January 2027	180	Patients ≥ 18 years with symptomatic CSDH or large asymptomatic CSDH after ≥ 6 weeks of failed conservative treatment	MMA embolisation performed within 72 h post-surgery or as a standalone treatment in non-surgical patients	Standard surgical treatment without embolisation or no intervention for non-surgical patients	Clinical or radiographic recurrence of CSDH within 6 months
ELIMINATE; MMAE in CSDH NCT04511572 [55]	Netherlands	To investigate the effect of MMA embolisation as an adjunct to surgical evacuation	Multicenter, open-label multicenter RCT	October 2025	170	Patients 50–90 years with symptomatic CSDH on imaging requiring surgery	MMA embolisation within 72 h after BHC	Only BHC	Recurrence requiring reoperation within 24 weeks after the intervention
MEMBRANE; MMAE after CSDH evacuation NCT05327933 [79]	Germany	To show that additional MMA embolisation reduces the risk of recurrent bleeding in patients with CSDH	Assessor-blinded, parallel RCT	October 2025	154	Patients ≥ 18 years who were operated on by one or more burr hole trepanations for primary CSDH	MMA embolisation using digital subtraction angiography	Standard of care	Recurrence of CSDH within 3 months
Efficacy of postoperative MMAE ACTRN12621000263897 [3]	Australia	To evaluate the effectiveness of postoperative MMAE in reducing recurrence rate, residual haematoma thickness, and improving functional outcomes in CSDH	Multicenter, asessor-blinded RCT	Unknown	106	Patients ≥ 18 years requiring surgical evacuation of symptomatic CSDH with a maximal thickness of ≥ 10 mm	MMA embolisation following burr-hole or craniotomy	Standard postoperative monitoring (no embolisation)	Symptomatic recurrence requiring reoperation within 3 months
MMAE as primary treatment for CSDH ACTRN12621000202864 [4]	Australia	To demonstrate that MMA embolisation can reduce the need for surgical evacuation in stable patients	Single-center RCT with blinding of the outcome assessor and statistician	December 2025	72	Patients ≥ 18 years with CSDH not requiring emergency surgical evacuation	MMA embolisation with Onyx liquid embolic agent	Observation with repeat CT brain at 6 weeks, 3 months, and 6 months	Symptomatic recurrent/residual CSDH that requires surgical evacuation at 6 weeks, 3 months, and 6 months
Endovascular embolisation for CSDH after surgery NCT04272996 [83]	USA	To evaluate whether MMA embolisation following surgical evacuation of CSDH reduces the rate of recurrence compared to surgical evacuation alone	Open-label, parallel RCT	Recruitment complete June 2024	60	Patients 18–90 years with CSDH requiring surgical evacuation with GCS > 6 and mRs < 5	Craniotomy followed by MMA embolisation	Ccraniotomy alone	Radiographic recurrence of CSDH within 3 months
MMAE to Treat CSDH NCT04095819 [98]	USA	To evaluate the safety and efficacy of a new, less-invasive procedure to treat CSDH	Open-label, pilot RCT	Unknown	50	Patients ≥ 18 years with symptomatic (acute-on) CSDH on imaging and ineligibile for conservative treatment	MMA embolisation	Surgical drainage through BHC or craniotomy	Change in size of SDH at 6 months
**Miscellaneous trials = 2**									
CSDH-LP; Low intracranial pressure treatment for CSDH NCT04607447 [37]	China	To explore the effectiveness and safety of low intracranial pressure treatment strategies for CSDH patients	Multicenter, assessor-blinded RCT	Recruitment complete 2023	160	Patients ≥ 14 years with CSDH ineligible for surgical treatment	ATO + DXM + low intracranial pressure: 1. Supine position for 16 h-18 h daily, head to affected side, 15–20 cm elevation of lower limbs 2. Abdominal belt compression	ATO + DXM	Subdural haematoma volume, MGS, mRS, and Extended Glasgow Outcome score at 3 months
Craniocervical manual lymphatic massage for CSDH ChiCTR2400093100 [24]	China	To investigate whether craniocervical manual lymphatic massage can promote haematoma absorption and improve meningeal lymphatic vessel drainage efficiency in conservative treatment of CSDH patients	Single-center, assessor-blinded RCT	June 2026	30	Patients 18–90 years with CSDH, mRS 1–3, and eligible for conservative treatment	Medical treatment + manual lymphatic drainage five times daily	Medical treatment (ATO 20 mg per dag + DXM placebo 0.75 mg three times daily for 2 weeks, two times daily for 1 week, and once daily for 1 week)	Neurological function before admission, at 2 weeks, 4 weeks, and 3 months And function of the meningeal lymphatic vessel before admission and at 2 weeks

*Trials addressing multiple topics are represented in multiple entries within the table

*ASA* American Society of Anesthesiologists, *ATO* atorvastatin, *BHC* burr hole craniostomy, *CSDH* chronic subdural haematoma, *CT* computed tomography, *DXM* dexamethasone, *GCS* Glasgow Coma Scale, *IV* intravenous, *MGS* Markwalder Grading Scale, *MLS* midline shift, *(pre-)mRS* (premorbid) modified Rankin Scale, *PROBE* Prospective Randomised Open, Blinded Endpoint, *RCT* randomised controlled trial, *TXA* tranexamic acid

Nearly 50% of recently published trials had a high risk of bias, primarily due to bias in the measurement of the outcome. The lowest risk of bias was observed in trials investigating perioperative management of CSDH. Detailed descriptions of the results from the risk of bias assessment using the RoB 2 tool are provided in the Supplementary Information (Online Resource 1).

### Steroid trials

In recent years, five clinical trials investigating corticosteroids have been published, four of which were previously identified in the 2019 review [54, 75, 76, 91, 121]. Among these, dexamethasone emerged as the most extensively studied corticosteroid. These studies hypothesized that corticosteroids may improve outcomes in CSDH through inhibition of inflammation and membrane development [34, 52].

The two largest studies on dexamethasone are the Dex-CSDH and DECSA trials [54, 76]. The Dex-CSDH trial assessed dexamethasone as an adjunct to surgery whilst the DECSA trial compared dexamethasone alone to surgery. Both trials observed that dexamethasone reduced recurrence rates but led to worse functional outcomes, measured on the modified Rankin Scale, and a higher rate of adverse events. The HEMACORT trial evaluated prednisone as an adjunct to surgery and observed a reduction in radiological recurrence as well, but with no clear clinical benefit and increased complication rates [91].

Taken together, these trials do not support the routine use of steroids as either an adjunct or alternative to surgery. At this moment there are three active trials investigating dexamethasone, particularly in combination with atorvastatin [17, 18, 108].

### Tranexamic acid trials

Tranexamic acid (TXA) has been hypothesized to reduce CSDH volume due to its anti-fibrinolytic effect [74]. Two recently published RCTs investigated TXA, as an adjunct to surgery, in reducing postoperative CSDH recurrence [128, 133]. Although neither trial showed a significant reduction in reoperation rates, both reported a significant reduction in haematoma volume, consistent with earlier findings in the literature [63, 81]. The lack of effect on reoperation rates, despite reductions in haematoma size, may be attributable to limited statistical power and methodological shortcomings, including high risk of bias of these studies. Consequently, larger trials with robust methodology are still warranted.

At present, seven trials investigating TXA in the context of CSDH are ongoing. Of these, three were identified in the previous review [82, 123, 125], while four are newly initiated [30, 31, 122, 124]. The previously identified trials include two evaluating TXA as stand-alone treatment (TRACS and TORCH) [82, 125], and one assessing TXA as an adjunct to surgery (TRACE) [123].

Among the newly initiated studies, two trials focus on TXA as stand-alone treatment [30, 124], whilst the TRACE-2 trial investigates patient-reported quality of life and CSDH recurrence with TXA either as stand-alone treatment or in combination with surgery [122]. Meanwhile, the TABASCO trial directly compares TXA and MMAE as adjuncts to surgery, aiming to clarify the comparative effectiveness of these two adjunctive strategies [31].

Collectively, these trials demonstrate that the role of TXA in CSDH treatment remains uncertain. Whether it can serve effectively as a stand-alone conservative therapy or as an adjunct to surgical intervention is the subject of continued investigation.

### Other pharmacological trials

Atorvastatin is a lipid-lowering agent with proposed anti-inflammatory and angiogenesis-regulating effects [69]. Its effects in the treatment of CSDH were previously investigated in the ATOCH trial, which found a statistically significant, though clinically modest, reduction in haematoma size of 12 mL between the atorvastatin and placebo groups [61]. Two recently published trials have also evaluated the role of atorvastatin in the treatment of CSDH [129, 134]. One trial assessed patients managed conservatively [134], while the other combined atorvastatin with low-dose dexamethasone following surgery [129]. Both studies reported significant reductions in haematoma volume and improvements in neurological outcomes, with the combination therapy demonstrating more pronounced effects. However, findings are of limited value due to small sample sizes and a high risk of bias.

There are ongoing studies assessing atorvastatin as a stand-alone treatment compared to placebo (REACH trial) [101], and again as a post-operative combined therapy with dexamethasone (ATOCH II, ADTCSDH, and ADELE trials) [17, 18, 108]. The REACH and ATOCH II trials, in particular, may have a meaningful impact on clinical practice due to their double-blind design and planned large sample sizes.

Traditional Japanese and Chinese herbal medicines are also under investigation. In Kampo medicine, the traditional herbal medicine in Japan, Goreisan and Saireito are commonly used. Goreisan is known for its hydragogue effect, while Saireito combines both hydragogue and anti-inflammatory properties. Both have been used empirically in the treatment of CSDH [127, 135]. Three completed trials examined Goreisan and/or Saireito, with two reporting no significant reduction in recurrence using Goreisan [45, 133], and the other demonstrating a significant reduction in recurrence with both agents [73]. However, all of these studies are at high risk of bias, primarily due to the absence of blinding or the lack of a placebo control group. Two ongoing trials are exploring traditional Chinese medicine: the CHARM trial evaluating HuoXue LiShui [38] and another trial evaluating the Peiyuan Huayu formula [23], both in conjunction with standard treatment. The CHARM trial [38] appears particularly promising, as it has a placebo-controlled design. HuoXue LiShui is a concept in traditional Chinese medicine which refers to the use of herbal treatments aimed at promoting blood circulation and reducing fluid stasis [132]. The Peiyuan Huayu formula, on the other hand, has demonstrated anti-angiogenic properties, which may be beneficial in managing CSDH [42]. While these herbal therapies may be widely accepted in certain regions, their broader adoption will depend on the reproducibility of findings outside Asia.

Given the high prevalence of antithrombotic use in the CSDH population [46, 66], optimizing antithrombotic therapy in CSDH patients is another key research focus. The recently published SECA trial found no significant increase in recurrence in CSDH patients that continued aspirin as opposed to those that discontinued aspirin [65]. This suggests early resumption of low-dose acetylsalicylic acid, or perhaps its complete continuation, may be possible without negative effects on effective CSDH treatment or recurrence. At present, the RELACS trial is an ongoing study that aims to investigate the optimal postoperative timing for restarting oral anticoagulants in CSDH patients with atrial fibrillation [105].

Taken together, these trials reflect an expanding interest in pharmacological strategies for CSDH across different treatment pathways. Importantly, more ongoing studies are multicenter, with larger sample sizes, and assessor-blinded, often alongside the use of a placebo control. These factors underscore the methodological limitations of earlier single-center and unblinded studies.

### Surgical trials

The optimal surgical management of CSDH has long been a subject of debate. While burr hole craniostomy (BHC) is generally considered the gold standard [15, 40, 93, 111], alternative surgical strategies such as craniotomy and twist-drill craniostomy (TDC) remain in clinical use. The COMPACT study [33], which was identified as ongoing in the 2019 review, compared these three operative modalities. Although it did not find a statistically significant difference in outcomes between the treatment arms, BHC was associated with the lowest recurrence rate (7.6% for BHC, 13.1% for craniotomy, And 19.5% for TDC) and a complication profile similar to the other treatment arms. BHC remains the most extensively studied surgical approach in recent years, and there has been additional focus on irrigation, drainage techniques and duration, and surgical cosmesis.

Two large Scandinavian trials (FINISH and SIC!) [11, 100] demonstrated that irrigation at body temperature significantly reduces operative recurrence rates, compared to irrigation at room temperature or no irrigation. An ongoing Japanese trial is currently investigating the use of artificial cerebrospinal fluid for irrigation [62], differing from the irrigation solutions used in the FINISH (saline) and SIC! (Ringer’s lactate) trials.

The method of postoperative drainage has also been investigated, comparing subdural drainage (SDD), the most commonly used technique, with subperiosteal drainage (SPD) [14, 97, 114]. All recent studies found SPD to be a safe and effective alternative to SDD. However, SPD did not meet the predefined non-inferiority margin in the cSDH-Drain-Trial, despite being associated with lower recurrence rates, fewer surgical site infections, and reduced drain misplacement [114]. More definitive conclusions may emerge from the ongoing SUPERDURA trial [2], which compares active SPD with passive SDD. Meanwhile, another ongoing study is evaluating the feasibility of using augmented reality to assist in subdural drain placement, potentially enhancing the safety and precision of standard SDD [87].

The previous review identified the CORRECT-SCAR trial [117], which assessed the use of burr hole covers to improve aesthetic outcomes. The study reported high overall patient satisfaction after BHC, without significant therapy benefit from the use of burr hole covers.

Other new studies have compared single versus double burr hole techniques and found no difference in clinical effectiveness, but a significantly shorter operating time with single burr hole procedures [107, 110]. An ongoing study is examining the efficacy of burr holes drilled in the frontal versus parietal bone, which may affect operative strategy [19].

### Perioperative management trials

Two large Danish trials assessed drainage duration following BHC [49, 60]. Shortening drainage time to 6 or 12 h was associated with increased recurrence, whereas no significant difference was observed between 24 and 48 h. These findings support a 24-h drainage period as a safe and efficacious drainage period. The ongoing ECHO trial further explores this topic by comparing exhaustive drainage to a fixed-time protocol, aiming to determine whether more intensive drainage with urokinase injections can reduce recurrence rates [103].

The GET-UP trial, identified in the previous review, demonstrated that early mobilisation (< 12 h after surgery, mean 5.5 h) was associated with fewer complications and lower CSDH recurrence compared to 48 h of bedrest, suggesting a potential shift in postoperative care standards [115].

The choice of anesthetic technique remains a subject of debate, with both general and locoregional anesthesia commonly used in CSDH surgery [1, 130]. The NEURANESTH trial compared these two approaches and found no significant difference in postoperative length of stay until medically fit for discharge, supporting flexibility in anesthetic choice [47]. However, since longer anesthesia durations have been associated with increased 1-year mortality in elderly patients with head injuries, locoregional anesthesia may offer a safer profile [131].

Finally, a novel concept is being evaluated in the BP-CSDH trial, which investigates whether postoperative posture (supine position with elevated lower body), aimed at managing intracranial hypotension, can reduce symptomatic recurrence [36].

### Middle meningeal artery embolisation (MMAE) trials

MMAE is an emerging treatment strategy for CSDH that has gained substantial research attention in recent years. While only two ongoing trials on MMAE were identified in the previous review, this number has increased to 21 currently registered and ongoing trials.

In addition to ongoing studies, five RCTs on MMAE have been published in recent years [29, 43, 70, 92, 113]. Notably, three of these – MAGIC-MT, EMBOLISE, and STEM – were simultaneously published in the New England Journal of Medicine in November 2024, prompting significant discussion within the neurosurgical and broader medical communities. All three trials evaluated MMAE as an adjunct to surgical treatment. The MAGIC-MT and STEM trials also included MMAE as a stand-alone treatment.

The MAGIC-MT trial [70], the largest of the three, investigated MMAE using Onyx for embolization. This study reported a reduction in serious adverse events in the embolisation group without significant difference in symptomatic recurrence or haematoma progression within 90 days between the embolisation group and usual-care group (both conservatively and surgically managed CSDH patients).

The EMBOLISE trial [29], also employing Onyx, demonstrated a significant reduction in reoperation rates in the embolisation and operative treatment arm compared to operative treatment alone.

The STEM trial [43] utilized Squid liquid embolization and defined treatment failure as a composite outcome including recurrent or residual CSDH (> 10 mm), reoperation or surgical rescue, disabling stroke, myocardial infarction, or death from a neurologic cause. The trial found that MMAE significantly reduced the risk of treatment failure in the embolisation group compared to the usual-care group (both conservatively and surgically managed CSDH patients). This effect was particularly driven by patients managed non-surgically, although the study was not sufficiently powered to draw definitive conclusions for this subgroup.

Considering these findings, the clinical role of MMAE in the management of CSDH remains uncertain. The variability in results may be due to heterogeneity in patient populations and differences in outcome definitions across studies. The trials have been met with critical responses in the literature, including a recent multidisciplinary consensus statement and several letters to the editor [10, 44, 58]. These critiques highlight methodological concerns and reflect the high risk of bias assessments assigned to these studies in the present review.

Most recently, the EMPROTECT trial [113] investigated MMAE using microparticles rather than non-adhesive liquid agents. The study focused on a CSDH patient population considered at high risk for recurrence. Although the trial observed effect estimates similar to those reported in other recent MMAE studies, it did not demonstrate a statistically significant reduction in recurrence rates at 6 months. It remains uncertain which patient population benefits most from this intervention.

Further research is warranted. The 21 ongoing trials on MMAE explore a range of approaches, including its use as an adjunct to surgery or as a stand-alone treatment. They also compare different embolic materials, such as adhesive and non-adhesive liquid agents, polyvinyl alcohol particles, and cyanoacrylate compounds. Despite variations in design, most of these studies share a common objective: to determine the clinical effectiveness of MMAE in treating CSDH.

## Discussion

This review builds upon the 2019 iCORIC systematic review by providing a detailed assessment of recently completed and ongoing RCTs in the field of CSDH. It offers a comprehensive overview of the therapeutic landscape, identifies overlap between studies, and suggests directions for future research to address persistent knowledge gaps. Findings from the RCTs presented in this review are crucial to reduce unwarranted practice variation at both international and intrahospital levels.

The recent publications of clinical practice guidelines in the United Kingdom and Denmark mark a significant milestone in the standardization of CSDH care. Several findings from this review are reflected in those recommendations. Notably, one example is the discouragement of dexamethasone following multiple trials reporting greater harm than benefit of the treatment [54, 76]. This reflects a clear shift from corticosteroids, previously the most studied intervention, to MMAE, which now dominates the research landscape.

This evolving focus suggests a parallel shift in clinical sentiment. Physicians should be less inclined to prescribe dexamethasone and may be more likely to consider MMAE as a potential adjuvant therapy. However, such shifts must be approached with caution. While the discouragement of routine dexamethasone use is supported by several trials, some findings suggest potential benefit in specific patient subgroups [6, 51]. Thus, while dexamethasone should not be part of routine care, further research into its targeted use, or at lower doses, may still hold value. Additionally, other pharmacological alternatives, such as tranexamic acid, are currently under investigation and may also prove beneficial in preventing surgery in vulnerable older patients with CSDH. Given limited resources and competing research priorities, a Value of Information (VOI) analysis [25] may help determine whether future research on the role of corticosteroids in CSDH treatment offers sufficient relevance relative to alternative therapies.

In contrast, MMAE has received increasing attention and is now the most studied intervention for CSDH. Although recent studies suggest that MMAE may be effective, its high resource cost and the limited evidence of clear clinical efficacy argue against its routine implementation in standard practice at this moment. Therefore, it has been proposed that MMAE should only be considered in carefully selected patients [10, 44, 58]. Additionally, the rapid expansion of research in this area raises concerns about potential saturation. Many ongoing studies share similar objectives and designs, which poses a risk of redundancy and inefficient resource use. Greater collaboration among research groups could consolidate efforts into fewer but larger, adequately powered trials.

As with any area of evidence-based medicine, the optimal treatment approach must be individualized to each patient based on the best available data. Continued research in CSDH is vital to expand the evidence base, but this expansion must be strategic. Uncoordinated proliferation of trials poses a risk of compromising overall research quality and diluting clinical impact.

This review has several notable strengths. First, it includes all relevant RCTs, both published and ongoing, providing a comprehensive overview of the current research landscape. By conducting a formal risk of bias assessment, we were able to identify and account for methodological concerns, while also highlighting studies with stronger internal validity and more reliable findings.

Nevertheless, some limitations should be acknowledged. A relatively large portion of included trials carry a high risk of bias, which limits the conclusions we can draw from them. This was especially the case for trials investigating pharmacological agents. This underscores the need for future RCTs to adopt rigorous designs that include placebo controls, power calculations, appropriate outcome selection and blinding where feasible, to enhance internal validity. Furthermore, the risk of publication bias due to trials that were abandoned or never initiated cannot be fully excluded. Thirteen trials were excluded because of unclear or discontinued trial status, as summarized in the Supplementary Information (Online Resource 1). Six trials reported discontinuation, typically for financial or logistical reasons, which would not in themselves introduce publication bias. Most of these involved novel research concepts, limiting their impact on the results summarized in this review. In contrast, seven trials were excluded because their estimated (or actual) completion date was more than three years before our search date, yet no publication or update was available. These trials appear to have been abandoned for unclear reasons, although the absence of registry updates does not necessarily confirm abandonment. To reduce the risk of missing trials for unknown reasons, we attempted to contact principal investigators. However, the lack of response leaves some uncertainty and the possibility of publication bias.

## Conclusions

In conclusion, the number of ongoing RCTs in CSDH has increased substantially in recent years, with a noticeable shift in research focus from corticosteroids to MMAE. The growing number of MMAE trials may indicate research saturation, emphasizing the need for consolidation into fewer, larger, and methodologically robust studies. Only through coordinated, high-quality research efforts can the field continue to progress toward optimal, evidence-based, and patient-centred care for individuals with CSDH.

## Supplementary Information

Below is the link to the electronic supplementary material.

ESM 1 Supplementary Material 1 (DOCX 930 KB)

## Data Availability

No datasets were generated or analysed during the current study.

## Abbreviations

*ATO*: Atorvastatin
*BHC*: Burr hole craniostomy
*CSDH*: Chronic subdural haematoma
*CT*: Computed tomography
*iCORIC*: International COllaborative Research Initiative on CSDH
*DXM*: Dexamethasone
*MMAE*: Middle meningeal artery embolization
*PRISMA*: Preferred Reporting Items for Systematic Reviews and Meta-Analyses
*RCT*: Randomised controlled trial
*RoB*: Risk of bias
*SDD*: Subdural drainage
*SPD*: Subperiosteal drainage
*TDC*: Twist drill craniostomy
*TXA*: Tranexamic acid
