# Supplementary material for: Updated systematic review of current randomised controlled trials in chronic subdural haematoma
Source: Acta Neurochir (Wien). 2025 Nov 6;167(1):288. doi: 10.1007/s00701-025-06683-5 (PMC12592311; doi:10.1007/s00701-025-06683-5)
Supplement: Supplementary file 1 — Supplementary Material 1 (DOCX 930 KB) [file 701_2025_6683_MOESM1_ESM.docx]

**Updated Systematic Review of Current Randomised Controlled Trials in Chronic Subdural Haematoma**

*R. Fakhry^a^, BSc; C. Yesildal^a^; J. Bartek^b, d^, MD, PhD; J. Duerinck^c^, MD, PhD; T.S.R. Jensen^d^, MD; J. Soleman^e^, MD, PhD; C. Iorio-Morin^f^, MD, PhD; C.M.F. Dirven^a^, MD, PhD; R. Dammers^a^, MD, PhD; E. Edlmann^g,^*, MD, PhD; D.C. Holl^a,^*, MD, PhD on behalf of the International Collaborative Research Initiative on Chronic Subdural Haematoma (iCORIC) study group*

*Shared senior authorship

1. Department of Neurosurgery, Erasmus Medical Center, Erasmus MC Stroke Center, Rotterdam, the Netherlands
2. Department of Neurosurgery, Karolinska University Hospital, Stockholm, Sweden and Department of Clinical Neuroscience, Karolinska institutet
3. Department of Neurosurgery, Universitair Ziekenhuis Brussel, Vrije Universiteit Brussel, Brussels, Belgium
4. Department of Neurosurgery, Copenhagen University Hospital, Copenhagen, Denmark
5. Department of Neurosurgery, University Hospital Basel, Basel, Switzerland
6. Division of Neurosurgery, Department of Surgery, Centre Hospitalier Universitaire de Sherbrooke, Sherbrooke, Canada
7. Department of Neurosurgery, South West Neurosurgical Centre, Plymouth, United Kingdom

**Corresponding author**
Rahman Fakhry, BSc
Erasmus MC, Department of Neurosurgery
Dr. Molewaterplein 40
3015 GD Rotterdam, the Netherlands
[r.fakhry@erasmusmc.nl](mailto:r.fakhry@erasmusmc.nl)

*Submitted to: Acta Neurochirurgica*

**Fig. S1** Traffic light plot of risk of bias assessment with the Cochrane RoB 2 tool for all recently published steroid trials


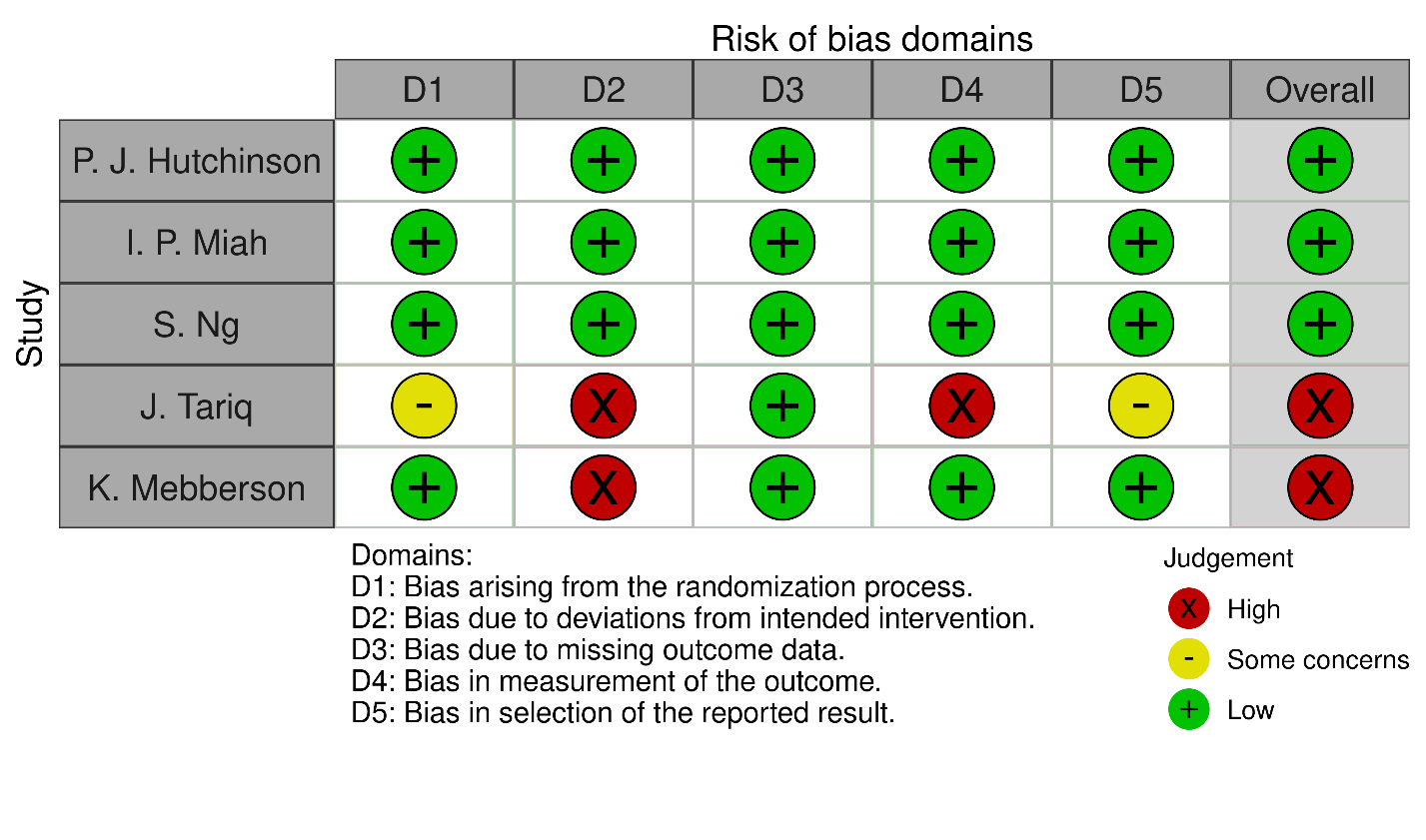


**Fig. S2** Traffic light plot of risk of bias assessment with the Cochrane RoB 2 tool for all recently published tranexamic acid trials


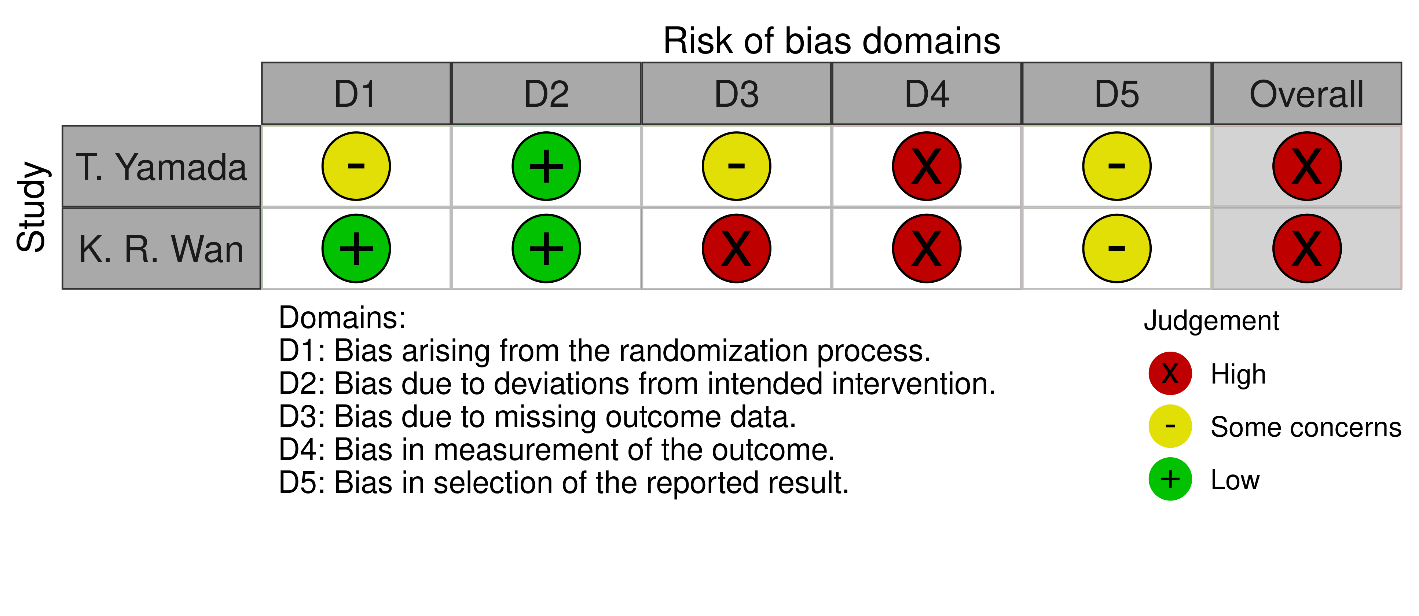


**Fig. S3** Traffic light plot of risk of bias assessment with the Cochrane RoB 2 tool for all recently published other pharmacological trials


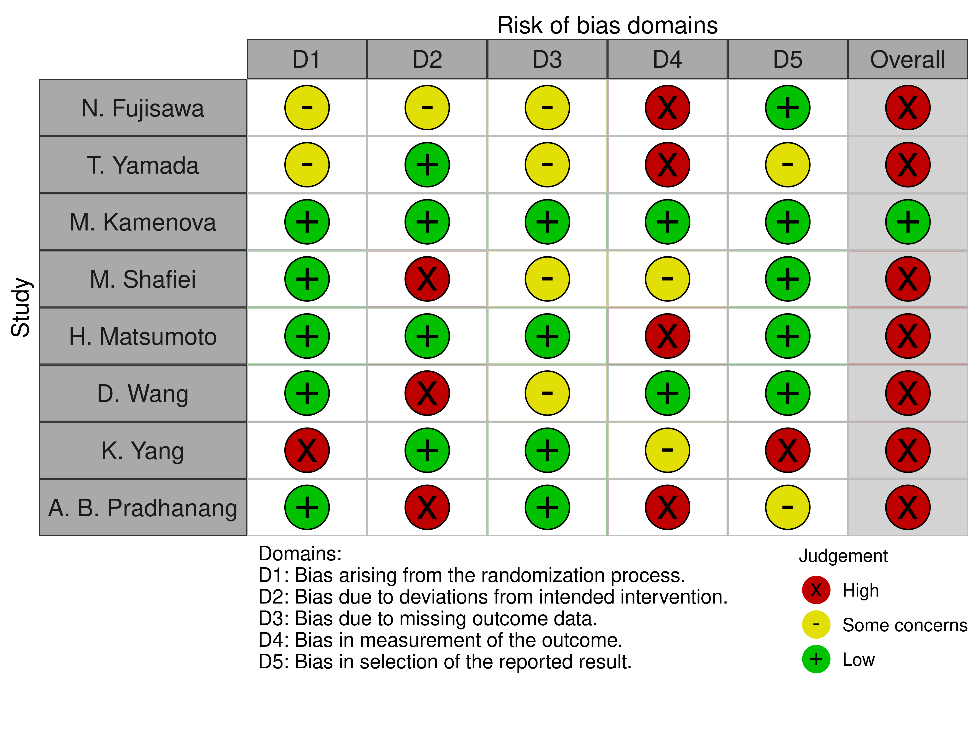


**Fig. S4** Traffic light plot of risk of bias assessment with the Cochrane RoB 2 tool for all recently published surgical trials


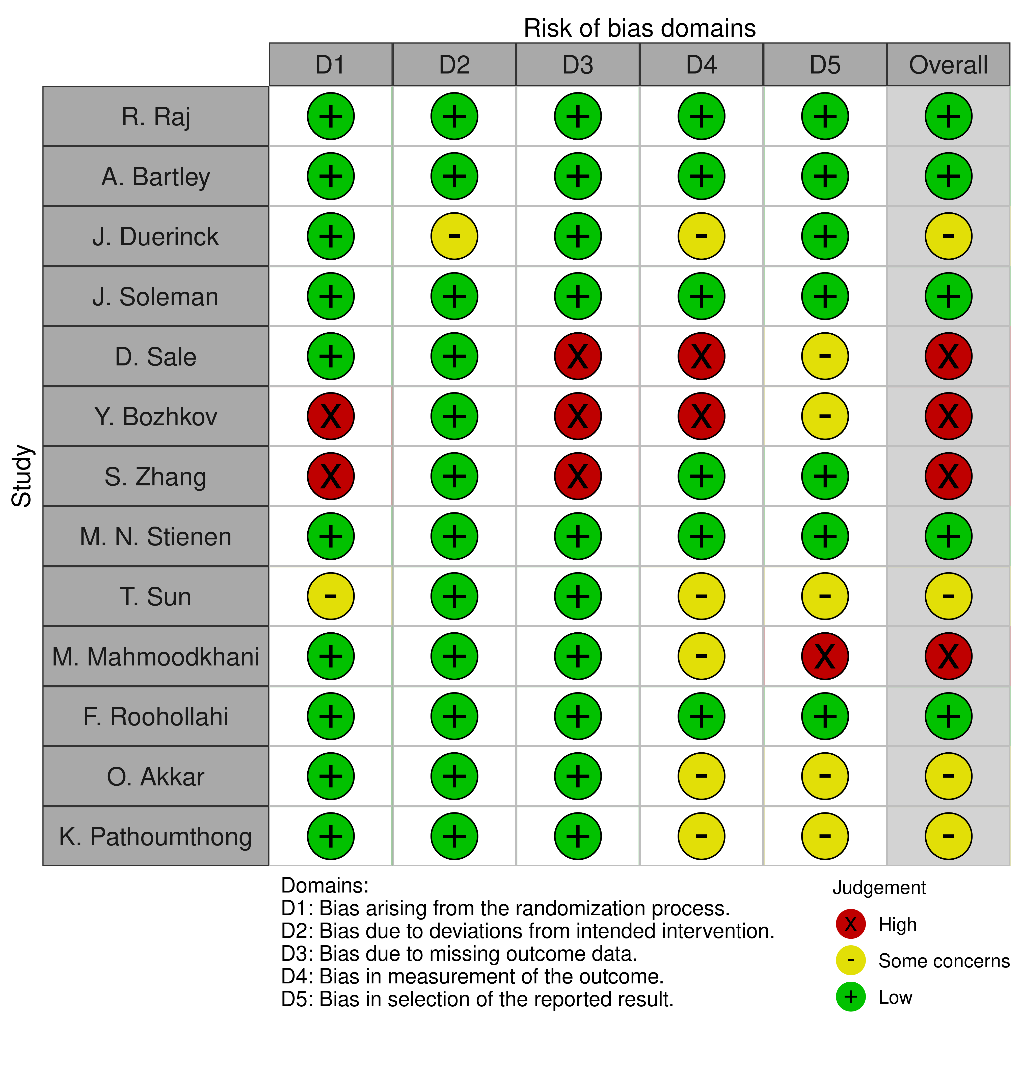


**Fig. S5** Traffic light plot of risk of bias assessment with the Cochrane RoB 2 tool for all recently published perioperative management trials


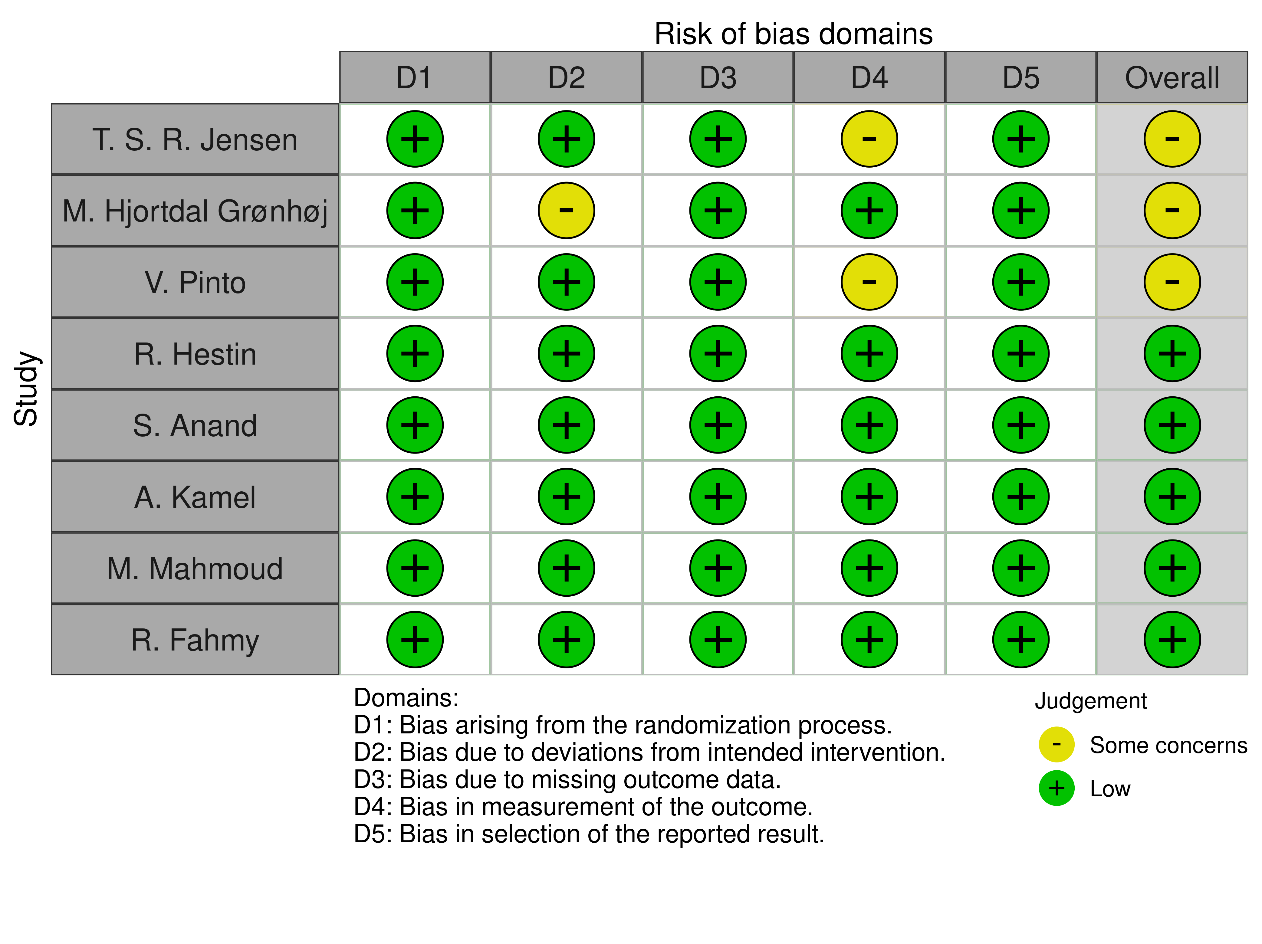


**Fig. S6** Traffic light plot of risk of bias assessment with the Cochrane RoB 2 tool for all recently published MMA embolisation trials


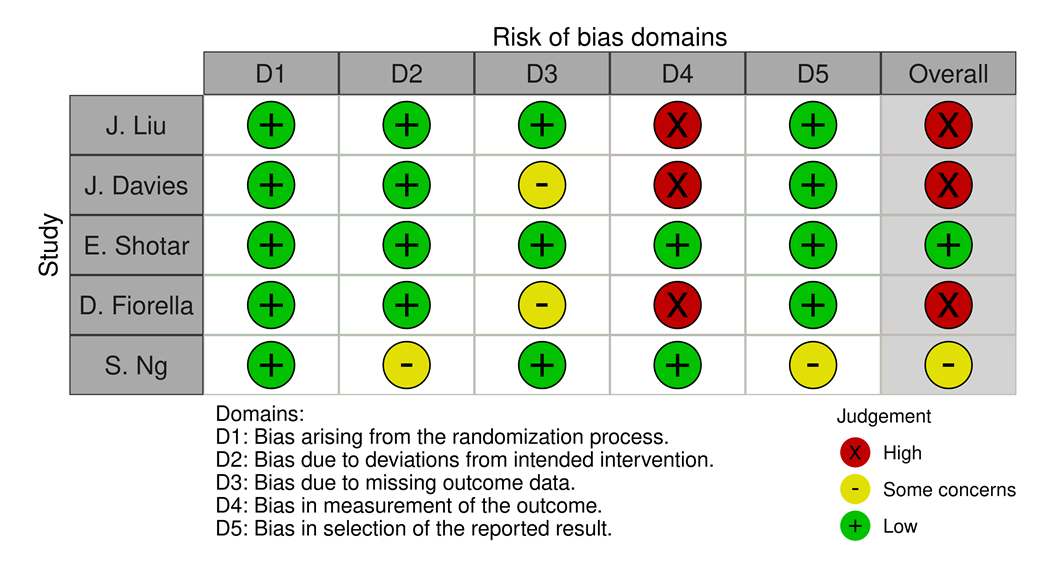


**Fig. S7** Traffic light plot of risk of bias assessment with the Cochrane RoB 2 tool for all recently published miscellaneous trials


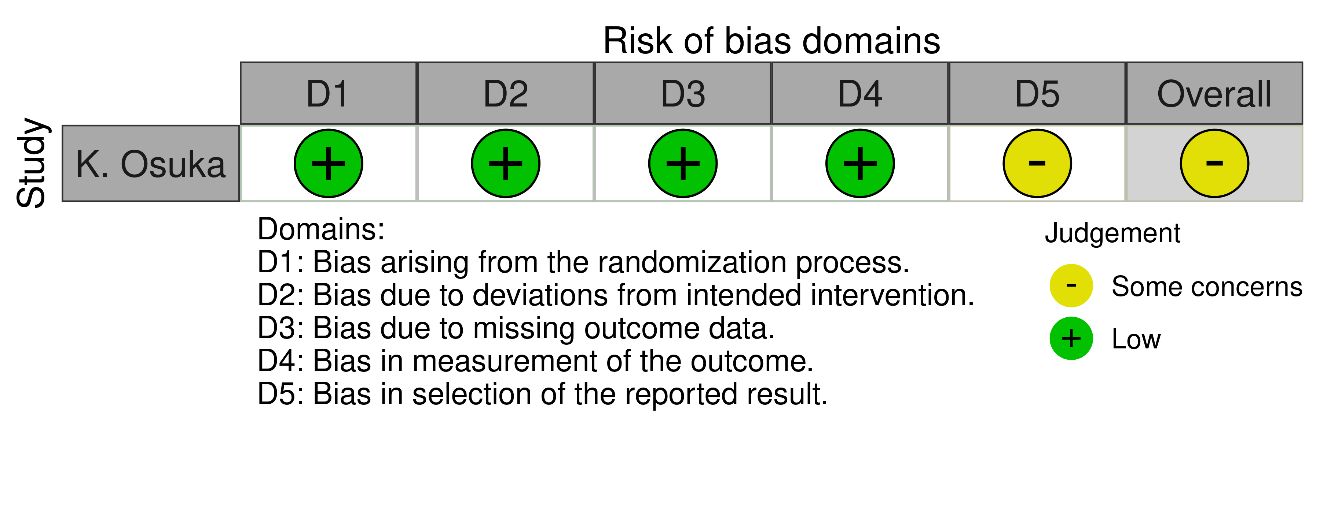


**Fig. S8** Summary plot of risk of bias assessment with the Cochrane RoB 2 tool for all recently published trials


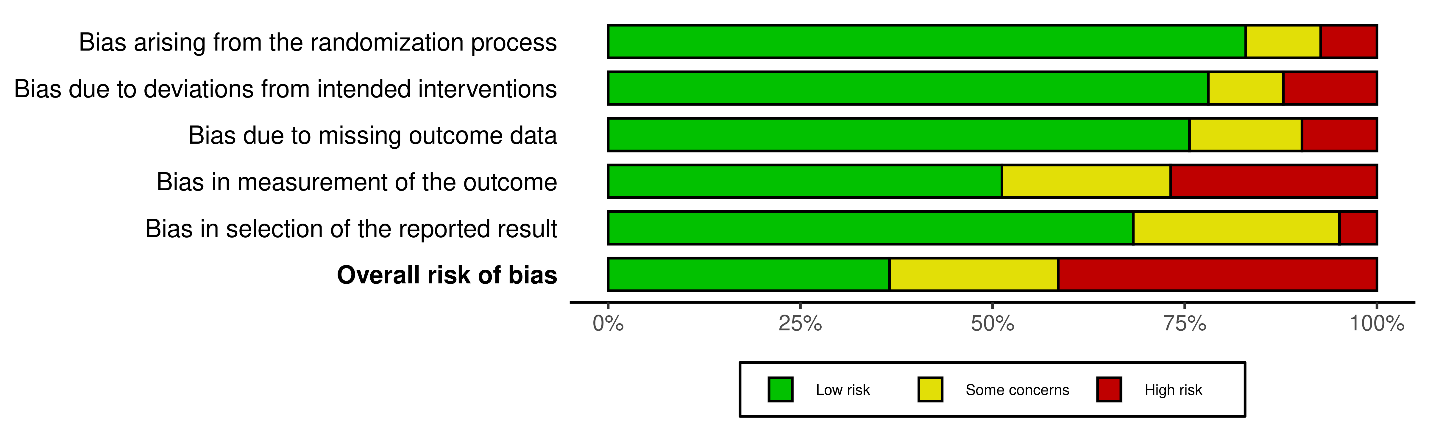


**Table S1** Overview of RCTs in CSDH with unknown or discontinued status, excluded from this review

| **Title** | **Country** | **Primary objective** | **Trial design** | **Estimated completion** | **N** | **Patients** | **Intervention** | **Comparison** | **Outcome** | **Reason for exclusion** |
| --- | --- | --- | --- | --- | --- | --- | --- | --- | --- | --- |
| **Steroid trials = 3** | | | | | | | | | | |
| [DRESH; DXM in reduction of reoperation rate for CSDH  EUCTR201100354442](https://www.clinicaltrialsregister.eu/ctr-search/search?query=2011-003544-42) [2] | Austria | To test the efficacy of 6 days postoperative DXM on reduction in the reoperation rate of CSDH | Multicenter, double-blind, placebo-controlled RCT | Unknown | 820 | Patients ≥ 25 years requiring surgical treatment of CSDH due to symptoms or radiological findings | DXM for 6 days, started within 48 h of surgery (16-16-12-12-8-4 mg/day) | Placebo as per DXM regimen | Reoperation rate within 12 weeks of primary surgery | Unknown trial status for > 3 years |
| [Management of CSDH using DXM  NCT02938468](https://clinicaltrials.gov/ct2/show/NCT02938468) [5] | Canada | To evaluate the efficacy and safety of DXM in management of CSDH | Single-center, open-label RCT | September 2021 | 326 | Patients ≥ 18 years with symptomatic CSDH, subacute or chronic on imaging | DXM for 21 days | Any surgical procedure for CSDH treatment | Surgical intervention in the DXM group or reoperation in the surgical group at 3 months | Unknown trial status for > 3 years |
| [SUCRE; Treatment of CSDH by corticosteroids](https://clinicaltrials.gov/study/NCT02650609)  [NCT02650609](https://clinicaltrials.gov/study/NCT02650609) [6] | France | To evaluate the efficacy of corticosteroids in CSDH patients without clinical or radiological signs of severity | Double-blind, placebo-controlled RCT | March 2020 (completed) | 202 | Patients ≥ 18 years with primary CSDH without clinical/radiological signs of severity | Methylprednisolone for 21 days:  < 60 kg = 48 mg/day (3 tablets)  60–80 kg = 64 mg/day (4 tablets)  > 80 kg = 80 mg/day (5 tablets) | Placebo as per methylprednisolone regimen | Delay of CSDH surgical treatment measured at 1 month | Unknown trial status for > 3 years |
| **Tranexamic acid trials = 1** | | | | | | | | | | |
| [Tocilizumab (TMAB) and TXA as adjuncts in CSDH surgery  NCT03353259](https://clinicaltrials.gov/study/NCT03353259) [7] | Norway | To evaluate if adjuvant treatment with tocilizumab (TMAB) or TXA reduces recurrence in operated CSDH | 3-arm parallel open-label RCT | September 2021 (completed) | 600 | Patients 55-100 years with CSDH requiring surgery | Surgery + TXA 1 g/day until haematoma disappearance  Or surgery + TXA 1 g/day + tocilizumab 162 mg/week until haematoma disappearance | Surgery only (burr hole craniostomy) | Recurrence requiring reoperation within 6 months | Unknown trial status for > 3 years |
| **Other pharmacological trials = 4** | | | | | | | | | | |
| [ATTAACH; Anticoagulation therapy timing in atrial fibrillation after CSDH  NCT05472766](https://clinicaltrials.gov/ct2/show/NCT05472766) [11] | Canada | To test the feasibility of comparing early versus delayed resumption of anticoagulation after CSDH diagnosis or surgery in a larger multicenter RCT | Pilot RCT with blinding of the outcome assessor | June 2025 | 120 | Patients ≥ 18 years with CSDH on imaging, eligible for surgical drainage, and on therapeutic anticoagulation prior to presentation | Early resumption of anticoagulation at day 30 after diagnosis of CSDH | Late resumption of anticoagulation at day 90 after diagnosis of CSDH | Recruitment rate within 1 year and implementation of study protocol until study completion | Discontinued |
| [GENESIS; Nonepileptic, stereotypical and intermittent symptoms in CSDH  NCT04759196](https://clinicaltrials.gov/show/NCT04759196) [9] | Canada | To compare Topiramate against Levetiracetam between the NESIS group (nonepileptic, stereotypical, and intermittent symptoms) and the non-NESIS group in CSDH patients | Multicenter, double-blind, placebo-controlled RCT | December 2024 | 56 | Patients ≥ 18 years with transient neurological symptoms in the context of CSDH and negative initial electroencephalogram | Topiramate 100 mg/day, with increases of 50 mg by week until efficacy, to a maximum of 200 mg/day in both the NESIS and non-NESIS group | Levetiracetam 1000 mg/day, with increase of 1000 mg die divided in two doses each week until efficacy, to a maximum of 3000 mg/day in both the NESIS and non-NESIS group | Difference in number of transient neurological symptoms reported within 6 months in patients in the NESIS group | Discontinued |
| [CACTIS; Curcumin/turmeric in CSDH recurrence  NCT03845322](https://clinicaltrials.gov/study/NCT03845322) [3] | USA | To evaluate the efficacy of curcumin in preventing a recurrence of CSDH following surgical evacuation | Single-center, double-blind, placebo-controlled pilot RCT | September 2020 | 48 | Patients ≥ 18 years with unliateral CSDH < 115 mL undergoing surgical evacuation | Curcumin 270 mg + black pepper extract 3 mg, three times daily for up to 60 days | Placebo as per curcumin regimen | Subdural hematoma volume change within 6 months | Discontinued |
| [TASD; Targeting spreading depolarization after CSDH surgery  NCT04966546](https://clinicaltrials.gov/ct2/show/NCT04966546) [10] | USA | To determine if a strategy of NMDA receptor antagonism can effectively reduce spreading depolarization and improve clinical recovery in CSDH patients | Double-blind, placebo-controlled pilot RCT | December 2025 | 20 | Patients ≥ 18 years undergoing CSDH surgery with eletrode strip placed during surgery and spreading depolarization detected within 48 hours of surgery | Memantine hydrochloride 20 mg/day for 7 days | Placebo as per memantine hydrochloride regimen | Spreading depolarizations and seizures within 5 days and extended Glasgow outcome scale score at 90 days | Discontinued |
| **Surgical trials = 2** | | | | | | | | | | |
| [DECiDE: DrainagE of ChronIc SubDural HEmatoma  NCT03053895](https://clinicaltrials.gov/study/NCT03053895?term=NCT03053895&rank=1) [4] | Canada | To demonstrate the effectiveness and safety of bedside drainage in CSDH patients | International, multicenter RCT with blinding of data analyst | February 2020 | 486 | Patients ≥ 18 years with symptomatic CSDH on imaging | Bedside twist drill technique | Operating room burr hole technique | Recurrence rate at 6 months | Unknown trial status for > 3 years |
| [SSS; surgical techniques for CSDH study  JPRN-UMIN000031033](https://trialsearch.who.int/?TrialID=JPRN-UMIN000031033) [13] | Japan | To compare drainage, irrigation or both combined on recovery and recurrence rate of CSDH | 3-arm parallel RCT with blinding of the outcome assessor | Unknown | 90 | Patients ≥ 20 years with symptomatic CSDH | * Only drainage  * Only irrigation  * Irrigation and drainage | None | Restlessness after surgery, recurrence and modified Rankin Scale score of 0 and 1 at 1 month | Unknown trial status for > 3 years |
| **Perioperative management trials = 1** | | | | | | | | | | |
| [GAS-CDE; General anaesthesia versus local anaesthesia  CTRI/2019/04/018544](https://www.ctri.nic.in/Clinicaltrials/pmaindet2.php?EncHid=MzI1NDE=&Enc=&userName=) [1] | India | To compare the effect of local versus general anaesthetic during CSDH surgery on postoperative cognitive change | Multicenter RCT with blinding of the outcome assessor | July 2020 | 250 | Patients 65–90 years with primary CSDH requiring evacuation and a Glasgow Coma Scale score of 15 | General anaesthesia with intravenous induction agent (propofol, thiopentone or etomidate), fentanyl and muscle relaxant and maintained on inhalation agent, with intubation and ventilation | Scalp block with lignocaine, adrenaline and bupivacaine plus supplemental sedation if require (propofol or dexmedetomidine) | Change in cognitive function as measured by a neuropsychologist from preoperatively to 24 h, 3 months and 1 year postoperatively | Unknown trial status for > 3 years |
| **MMAE trials = 1** | | | | | | | | | | |
| [ENCLOSURE; Endovascular embolization of CSDH after surgery  NCT05220826](https://clinicaltrials.gov/show/NCT05220826) [12] | Spain | To analyze the efficacy of and safety of early postsurgical MMA embolisation in reducing the risk of CSDH recurrence | Multicenter, open-label RCT with blinding of the outcome assessor | December 2023 | 280 | Patients with symptomatic CSDH, MLS > 5 mm, or haematoma thickness > 10 mm, and surgery planned in 72 hours | Surgical drainage plus early MMA embolisation within 72 hours of surgical evacuation | Surgical drainage | Recurrence of CSDH within 6 months | Discontinued |
| **Miscellaneous trials = 1** | | | | | | | | | | |
| [BANISH; Postoperative balloninflation after CSDH evacuation  NCT04060186](https://clinicaltrials.gov/show/NCT04060186) [8] | Germany | To evaluate the efficacy of balloninflation after CSDH surgery in reducing the risk of recurrence | Multicenter, open-label RCT | February 2022 | 200 | Patients ≥ 18 years surgically treated for CSDH with a subdural drain | Inflaction of a ballon made by a handglove 2-3 times/hour for 12 hours after CSDH surgery | No balloninflation after CSDH surgery | Recurrence requiring reoperation within 3 months | Discontinued |

*Abbreviations: CSDH, chronic subdural haematoma; DXM, dexamethasone; TXA, tranexamic acid.*

**References**

1. CTRI/2019/04/018544 (2019) Comparison of general anaesthesia and local anesthesia on post-operative cognitive change in elderly patients undergoing neurosurgery (trans: Dr Smita VY, Dr Sandhya MSA, Approved, Human Ethical Committee GMcTK, Institutional Ethics Committee SECRIKLRR).

2. Emich S, Richling B, McCoy MR, Al-Schameri RA, Ling F, Sun L, Wang Y, Hitzl W (2014) The efficacy of dexamethasone on reduction in the reoperation rate of chronic subdural hematoma--the DRESH study: straightforward study protocol for a randomized controlled trial. Trials 15:6. doi:10.1186/1745-6215-15-6

3. NCT03845322 (2019) Curcumin/Turmeric as a Treatment for Patients With Subdural Hematomas Recurrence (trans: University of New Mexico N).

4. Nct (2016) Bedside Versus Operating Room Burr-Hole Drainage of Chronic Subdural Hematoma (DECIDE). <https://clinicaltrialsgov/show/NCT03053895>

5. Nct (2016) Mgt of Chronic Subdural Hematoma Using Dexamethasone. <https://clinicaltrialsgov/show/NCT02938468>

6. Nct (2016) Treatment of Chronic Subdural Hematoma by Corticosteroids. <https://clinicaltrialsgov/show/NCT02650609>

7. Nct (2017) Tocilizumab (RoActemra) and Tranexamic Acid (Cyklokapron) Used as Adjuncts to Chronic Subdural Hematoma Surgery. <https://clinicaltrialsgov/show/NCT03353259>

8. Nct (2019) Postoperative Balloninflation After Evacuation of cSDH. <https://clinicaltrialsgov/show/NCT04060186>

9. Nct (2020) Generating Evidence on NonEpileptic, Stereotypical and Intermittent Symptoms (NESIS) in Chronic Subdural Hematomas. <https://clinicaltrialsgov/show/NCT04759196>

10. Nct (2021) Targeting Spreading Depolarization After Chronic Subdural Hematoma Surgery (TASD). <https://clinicaltrialsgov/ct2/show/NCT04966546>

11. Nct (2022) Anticoagulation Therapy Timing in Atrial Fibrillation After Acute and Chronic Subdural Hematoma. <https://clinicaltrialsgov/ct2/show/NCT05472766>

12. Nct (2022) Endovascular Embolization of Chronic Subdural Hematomas After Surgery. <https://clinicaltrialsgov/show/NCT05220826>

13. Umin (2018) Surgical techniques for chronic subdural hematoma study. <https://trialsearchwhoint/Trial2aspx?TrialID=JPRN-UMIN000031033>
